# A proteomics approach for the identification of cullin-9 (CUL9) related signaling pathways in induced pluripotent stem cell models

**DOI:** 10.1371/journal.pone.0248000

**Published:** 2021-03-11

**Authors:** Natalya A. Ortolano, Alejandra I. Romero-Morales, Megan L. Rasmussen, Caroline Bodnya, Leigh A. Kline, Piyush Joshi, Jon P. Connelly, Kristie L. Rose, Shondra M. Pruett-Miller, Vivian Gama

**Affiliations:** 1 Department of Cell and Developmental Biology, Vanderbilt University, Nashville, Tennessee, United States of America; 2 Department of Cell & Molecular Biology, St. Jude Children’s Research Hospital, Memphis, Tennessee, United States of America; 3 Center for Advanced Genome Engineering, St. Jude Children’s Research Hospital, Memphis, Tennessee, United States of America; 4 Department of Biochemistry, Vanderbilt University, Nashville, Tennessee, United States of America; 5 Vanderbilt MSRC Proteomics Core, Nashville, Tennessee, United States of America; 6 Vanderbilt Center for Stem Cell Biology, Nashville, Tennessee, United States of America; 7 Vanderbilt Brain Institute, Nashville, Tennessee, United States of America; University of Colorado Boulder, UNITED STATES

## Abstract

CUL9 is a non-canonical and poorly characterized member of the largest family of E3 ubiquitin ligases known as the Cullin RING ligases (CRLs). Most CRLs play a critical role in developmental processes, however, the role of CUL9 in neuronal development remains elusive. We determined that deletion or depletion of CUL9 protein causes aberrant formation of neural rosettes, an *in vitro* model of early neuralization. In this study, we applied mass spectrometric approaches in human pluripotent stem cells (hPSCs) and neural progenitor cells (hNPCs) to identify CUL9 related signaling pathways that may contribute to this phenotype. Through LC-MS/MS analysis of immunoprecipitated endogenous CUL9, we identified several subunits of the APC/C, a major cell cycle regulator, as potential CUL9 interacting proteins. Knockdown of the APC/C adapter protein FZR1 resulted in a significant increase in CUL9 protein levels, however, CUL9 does not appear to affect protein abundance of APC/C subunits and adapters or alter cell cycle progression. Quantitative proteomic analysis of CUL9 KO hPSCs and hNPCs identified protein networks related to metabolic, ubiquitin degradation, and transcriptional regulation pathways that are disrupted by CUL9 deletion in both hPSCs. No significant changes in oxygen consumption rates or ATP production were detected in either cell type. The results of our study build on current evidence that CUL9 may have unique functions in different cell types and that compensatory mechanisms may contribute to the difficulty of identifying CUL9 substrates.

## Introduction

Post-translational modifications critically regulate protein function, subcellular localization, structure, and turnover. Ubiquitination is a post-translational modification that primarily signals for protein turnover accounting for 80% of all protein degradation in cells [1]. However, ubiquitin can signal for more than proteasomal degradation, depending on the type of linkages within a polyubiquitin chain [2, 3]. Ubiquitin modification of a given substrate is catalyzed by three enzymes: E1 (activating enzyme), E2 (conjugating enzyme), and E3 (ubiquitin ligase). The E1 activates ubiquitin in an ATP dependent manner. Adenylated ubiquitin forms a thioester bond with the catalytic cysteine in the E1 active site and is then moved to the E2 via a thioester transfer. The E2-ubiquitin conjugate is recruited to an E3 ubiquitin ligase which aids or directly catalyzes ubiquitin thioester transfer to a specific residue, often a lysine, on the targeted substrate. The largest family of E3 ubiquitin ligases is the Cullin Ring Ligase (CRL) family.

CRLs function as multi-subunit complexes ubiquitinating a variety of substrates. The central part of a CRL is the cullin protein, a hydrophobic scaffolding protein that recruits a RING binding protein (Rbx1 or Rbx2) through the homologous cullin domain. CRL function also requires a covalent modification by the ubiquitin-like NEDD8 at a conserved lysine residue within the cullin scaffold. Each cullin contains a unique set of domains at their N-terminal that mediate substrate adapter binding. While there are eight cullins, there are numerous possible CRLs based on the recruited adapter proteins. Considering the ubiquitous nature of CRLs, they contribute to the regulation of nearly every cellular process. In fact, mouse knockout models of most members of the CRL family result in embryonic or neonatal lethality often as a result of aberrant cell cycle progression [4–8]. However, CRL family member CUL9 is an outlier.

CUL9 is considered a unique CRL, as it contains an additional RING between RING (RBR) domain at its C-terminus and has no identified adapter proteins [9–11]. CUL9 KO mice are viable, and only a few phenotypes have been reported, providing limited insight into its function [12–14]. Studies at the cellular level, including our own, identified a role for CUL9 in promoting survival in post-mitotic neurons and various cancer cell lines. CUL9 promotes ubiquitin mediated proteasomal degradation of cytochrome *c* in neurons and human brain tumor cell lines, and survivin in human cancer cells [9, 10]. With only two identified substrates, the mechanism of CUL9 ubiquitination is not known. Considering CUL9 has two potential ubiquitin catalytic domains, further characterization of CUL9 function and regulation is needed.

In view of the importance of CRLs in cell cycle and early development, human pluripotent stem cells (hPSCs) (denoting human embryonic stem cells and human induced pluripotent stem cells) may provide an advantageous biological system for characterizing the function of CUL9 in neuronal development. Indeed, our data indicates that CUL9 is required for normal neuronal differentiation. Differentiation of CUL9 KO embryoid bodies (EBs), 3D aggregates formed from hPSCs cultured in suspension, to neural rosettes, a developmental structure likened to organized neuroepithelial cells in the neural tube, resulted in phenotypic abnormalities. In this study, we use hPSCs and human neural progenitor cells (hNPCs) to identify CUL9 interacting proteins to screen as potential substrates and regulators in pluripotency and neuronal differentiation.

Our results indicate that CUL9 interacts with the anaphase promoting complex/cyclosome (APC/C) subunit APC7 in both hPSCs and hNPCs. Interaction and cross regulation between two CRLs is relatively common. In fact, CUL9 dimerizes with another CRL, CUL7. Although the dimerization is not necessary for its function, one study demonstrates CUL7 regulates CUL9 protein levels [13, 15]. Additionally, CUL1 and APC/C regulate one another to promote G1 progression and entry into the S-phase [16]. We show evidence that the APC/C and CUL9 may interact to regulate CUL9 protein stability, but not cell cycle progression.

We employed quantitative proteomics and identified significantly altered levels of separate sets of metabolic and transcriptional regulation proteins in hPSCs and hNPCs, providing evidence for a potential switch in CUL9 regulation during neuronal differentiation. Our results highlight the importance of integrating proteomic and functional approaches to understanding CUL9 function in human neuronal differentiation.

## Results

### CRISPR/Cas9 CUL9 KO iPSC clones are pluripotent

To explore the role of CUL9, we used hPSCs as a model. We first generated a CUL9 knockout cell line in human induced pluripotent stem cells (hiPSCs) using the CRISPR/Cas9 system (S1A–S1C Fig). Two clones containing a single base pair insertion were selected for downstream analysis. An unedited clone was also identified and used downstream as a control (isogenic WT). Western blotting was used to validate the loss of CUL9 protein expression in both knockout clones (S1C Fig). Metaphase spread analysis was used to validate that all isogenic clones had a normal karyotype (S2 Fig). Previous studies demonstrate that CUL9 heterodimerizes with another CRL known as CUL7 [10, 15]. Although CUL9 function does not require heterodimerization with CUL7, the two are highly homologous, and we wanted to ensure CUL7 protein levels would not increase to compensate for loss of CUL9. We determined by Western blotting analysis that CUL7 levels are not significantly changed in CUL9 KO iPSCs (S1C Fig).

**Fig 1:**
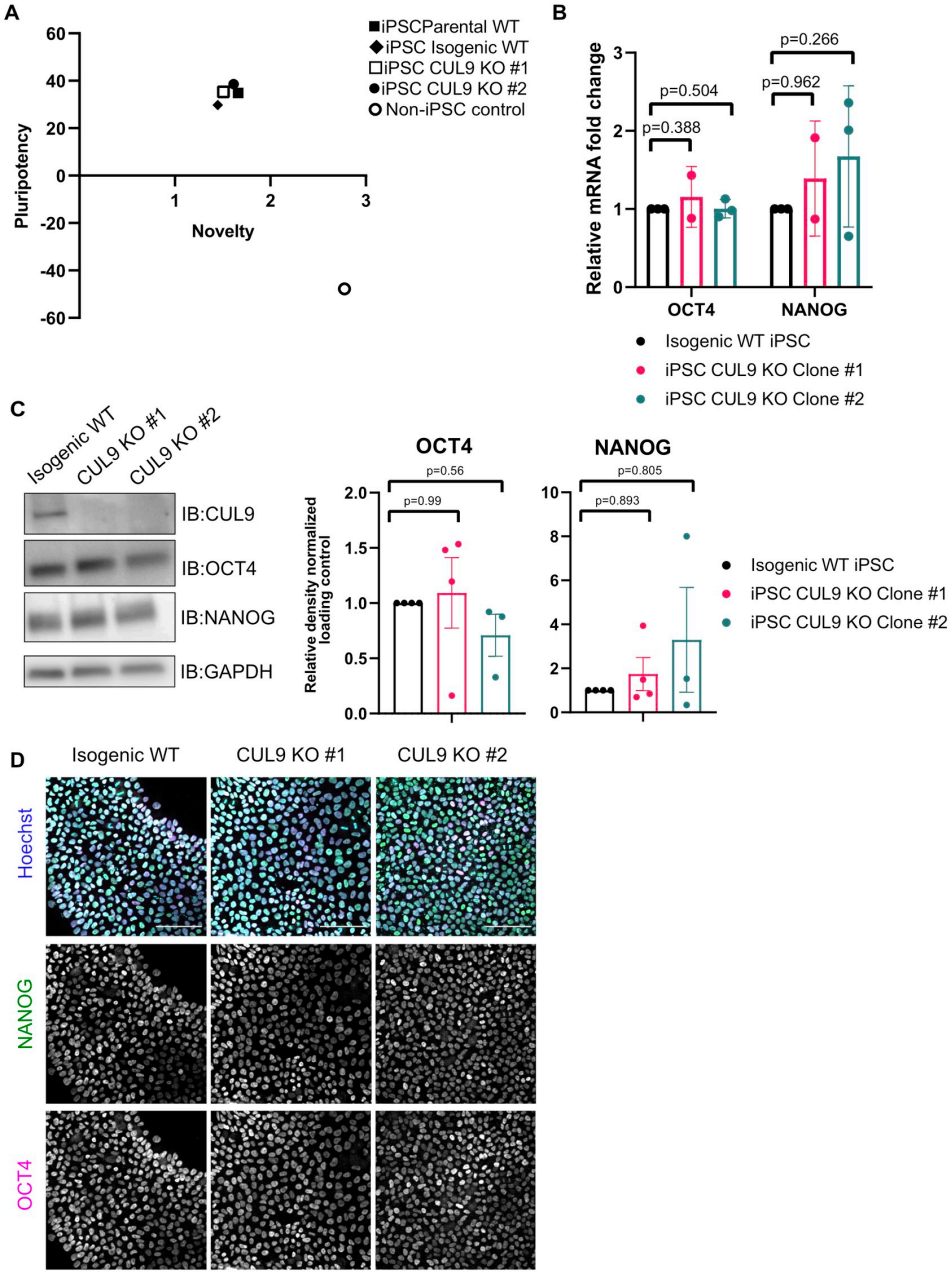
CUL9 KO hPSCs normally express key pluripotency genes. (**A**) CUL9 KO clones have expected global gene expression profile of pluripotent cell lines. Pluritest (Thermo) is a gene expression profile assay. Pluripotency score measures the presence of metagenes within the data set that separate pluripotent samples from non-pluripotent samples. Novelty score measures how well the gene expression pattern of samples fits expected profile. (**B**) RNA isolated from WT and CUL9 KO iPSCs were analyzed by RT-qPCR. Error bars +/- SD. iPSC. n = 3. (**C**) CUL9 KO cells express normal protein levels of key pluripotency markers as determined by Western Blotting. Isogenic WT and Clone #1 n = 4; Clone #2 n = 3; mean +/- SEM; Analysis done using student’s t-test, α = 0.05. (**D**) Key pluripotency markers are appropriately localized and expressed in CUL9 KO cells. hPSCs were fixed with 4% PFA and stained with key transcription factors NANOG (red, Alexa 546) and OCT4 (purple, Alexa 647), and Hoechst, marking nuclei (blue). Images acquired on Nikon Spinning Disk; 20X objective; scale bar = 100 μM.

Next, we assessed the effects of CUL9 deletion on the pluripotency of hiPSCs. We initially tested this using a global gene expression profile assay known as the PluriTest Assay [17]. Briefly, the gene expression profile of each clone was compared to a reference data set containing over 450 genome wide transcriptional profiles of validated pluripotent cells. The pluripotency score (y-axis) quantifies the presence of metagenes within the data set that separate pluripotent cells from non-pluripotent cells. The novelty score (x-axis) measures how well the gene expression pattern of the cell in question matches the expected profile. Compared to a non-iPSC control, our parental wild-type, isogenic wild-type, and CUL9 KO iPSCs have the expected transcriptional profile of a pluripotent cell (Fig 1A–1D). To complement the PluriTest assay, we examined the mRNA and protein level expression of key pluripotency factors OCT4 and NANOG. In both CUL9 KO clones, there were no significant changes in expression levels of OCT4 or NANOG as determined by RT-qPCR and Western blotting (Fig 1B and 1C). Additionally, confocal microscopy analysis of immunostained hiPSCs showed that OCT4 and NANOG localized to the nucleus in all cell types and are expressed at similar levels (Fig 1D). Based on our results, we concluded that the CUL9 KO clones remain pluripotent.

### CUL9 does not target cytochrome *c* for degradation

We previously reported that CUL9 promotes survival in post-mitotic neurons and neuroblastoma cells [9]. During apoptosis, cytochrome *c* is released from mitochondria, initiating the caspase cascade. Although this was previously considered the “point of no return” in apoptosis, we showed that CUL9 targets cytochrome *c* for ubiquitin mediated degradation following its release from the mitochondria. We hypothesized that cytochrome *c* would not be a CUL9 substrate as hPSCs are highly sensitive to apoptosis [18]. To test our hypothesis, we treated control and CUL9 KO cells with 1uM of the DNA damaging agent etoposide for 3 hours; then, we measured cell survival by examining caspase activity. CUL9 KO cells did not show differences in caspase 3/7 activity compared to control cells (S3 Fig). We further validated that CUL9 does not directly target cytosolic cytochrome *c* for degradation by examining cytochrome *c* release from mitochondria following a four hour treatment with 1μM etoposide in the presence of pan-caspase inhibitor QVD-OPh in control and CUL9 KO cells (S4 Fig). In both the control and KO cells, cytochrome *c* was released from the mitochondria into the cytosol after four hours of treatment at the same level. These data indicate that cytochrome *c* is not directly degraded by CUL9 in hPSCs, and that these cells rapidly initiate apoptosis in response to double stranded breaks as previously shown [18]. Additionally, we show that cytochrome *c* is likely targeted for proteasomal degradation in hPSCs as reported in neuronal cells [9] as cytochrome *c* levels increase with additional treatment of proteasome inhibitor bortezomib in both cell lines, though at lower levels in CUL9 KO cells (S4 Fig).

### CUL9 KO iPSC clones differentiate to hNSCs and hNPCs

**Fig 2:**
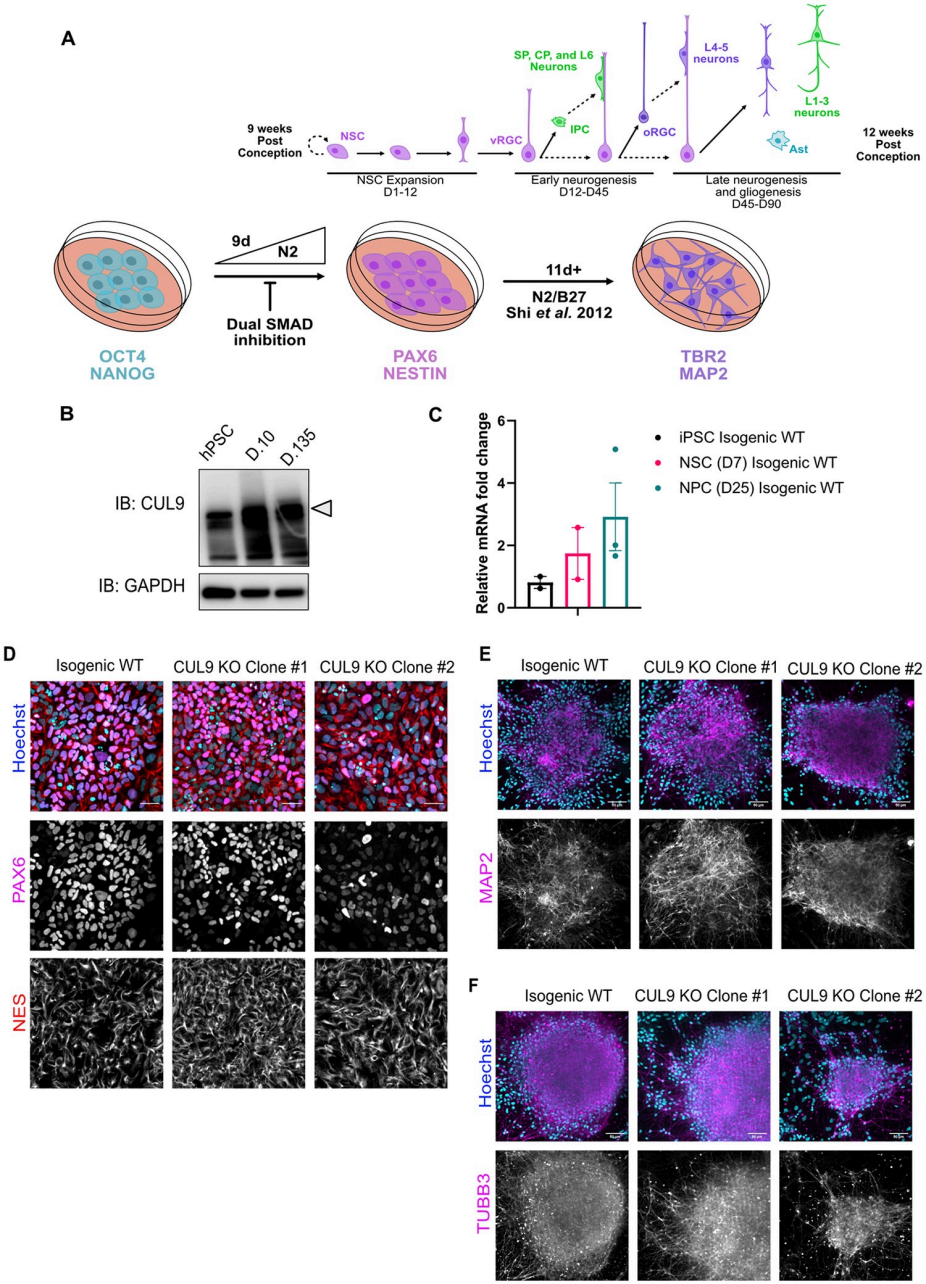
CUL9 KO clones differentiate to hNPCs and early born cortical neurons. (**A**) Protocol for neuronal differentiation of hPSCs. Diagram above *in vitro* differentiation roughly correlates to *in vivo* corticogenesis with differentiation protocol. Exact cell population at stages of differentiation often varies between differentiations. NPC = neuroepithelial progenitor cells; PP = preplate; vRGC = ventricular radial glia cells; IPC = intermediate progenitor cells; SP = subplate; CP = cortical plate; oRGC = outer radial glia cells; Ast = astrocyte (**B**) CUL9 protein levels increase over the course of cortical neuron differentiation. Western blot analysis of lysates obtained from WT neurons differentiated for ten days and 135 days show gradual increase in CUL9 protein levels compared to WT hPSCs. APC7 levels increase during initial neuronal induction, and then remain relatively stable throughout maturation. n = 3. (**C**) CUL9 mRNA levels increase over the course of cortical differentiation. RNA isolated from iPSCs, D7 cortical differentiation, and D25 cortical differentiation were analyzed by RT-qPCR. Error bars +/- SD. iPSC and D7 n = 2. D25 n = 3. Key hNPC (**D**) and neuronal markers (**E**) are appropriately localized and expressed in CUL9 KO cells. hNSCs were fixed with 4% PFA and stained with key transcription factor PAX6 (red, Alexa 546) and NESTIN (purple, Alexa 647), and Hoechst, marking nuclei (blue). hNPCs were fixed and stained with neuronal markers MAP2 (purple, Alexa 647) and TUBB3 (purple, Alexa 647). Images acquired on Nikon Spinning Disk; 20X objective; scale bar = 50 μM. NES = Nestin; TUBB3 = Beta-3-tubulin.

CUL9 is highly expressed in the brain, particularly in the cerebral cortex, based on RNA expression data obtained from human tissue samples (Human Protein Atlas) [19]. Transcriptome analysis of *in vitro* hPSC cortical differentiation revealed that CUL9 mRNA levels increase over the course of human cortical differentiation, most substantially during the neural induction phase when neural precursors form (identified using CORTECON database) [19]. We validated this induction at the protein level (Fig 2A–2C). Based on this new finding, we focused our study on characterizing the effects of CUL9 deletion in the first 25 days of cortical differentiation of hPSCs (Fig 2A). During the first ten days of cortical differentiation, N2 media is gradually increased during dual SMAD inhibition; this phase is known as neuralization. At this phase, neuroepithelial-like cells (which we have termed human Neural Stem Cells (hNSCs)) expressing neural stem cell markers like PAX6 and NESTIN begin to form. After day ten, N2/B27 media is used to maintain the cells, and over time the cells differentiate further [20]. Around day 25, the cells are a mixture of intermediate progenitor cells and immature cortical neurons (which we have termed human Neural Progenitor Cells (hNPCs)). These cells are highly migratory permitting them to form the cortical layers in an inside outside fashion. Cells at this phase express neuron specific microtubules and associated proteins including microtubule associated protein (MAP2) and beta-3 tubulin (TUBB3) [21, 22]. They also express transcription factors present in radial glia cells and intermediate progenitor cells like Empty Spiracles Homeobox 2 (EMX2), and those present in early born neurons like T-box brain 1 (TBR1) [23, 24]. Beyond day 25, the cells will form neurons in the expected order starting with deep-layer neuron populations followed by upper-layer neuron populations and astrocytes.

Both CUL9 KO iPSC clones successfully differentiated to hNSCs expressing neural stem cell transcription factor PAX6 and microtubule associated protein NESTIN (Fig 2D; S5B–S5D Fig). The clones further differentiated to hNPCs at day 25 and expressed microtubule markers TUBB3 and MAP2 as well as transcription factors EMX2 and TBR1 (Fig 2E; S6 Fig). Although there are little significant changes in protein or mRNA expression of neuronal markers at day seven or day 25 of CUL9 KO differentiated cells, substantial variability in expression is observed between replicates (S5B–S5D and S6 Figs). This clonal variability could indicate that CUL9 deficiency generates genomically unstable cell lines. A confounding factor is the variability between cortical differentiations using this method as previously reported [25]. To delineate if the observed variability in expression is a phenotype or a result of the differentiation method used, we employed 3D differentiation methods which have been demonstrated to be highly reproducible at differentiating neural cell populations [26].

### Deletion of CUL9 results in abnormal embryoid body and neural rosette formation

**Fig 3:**
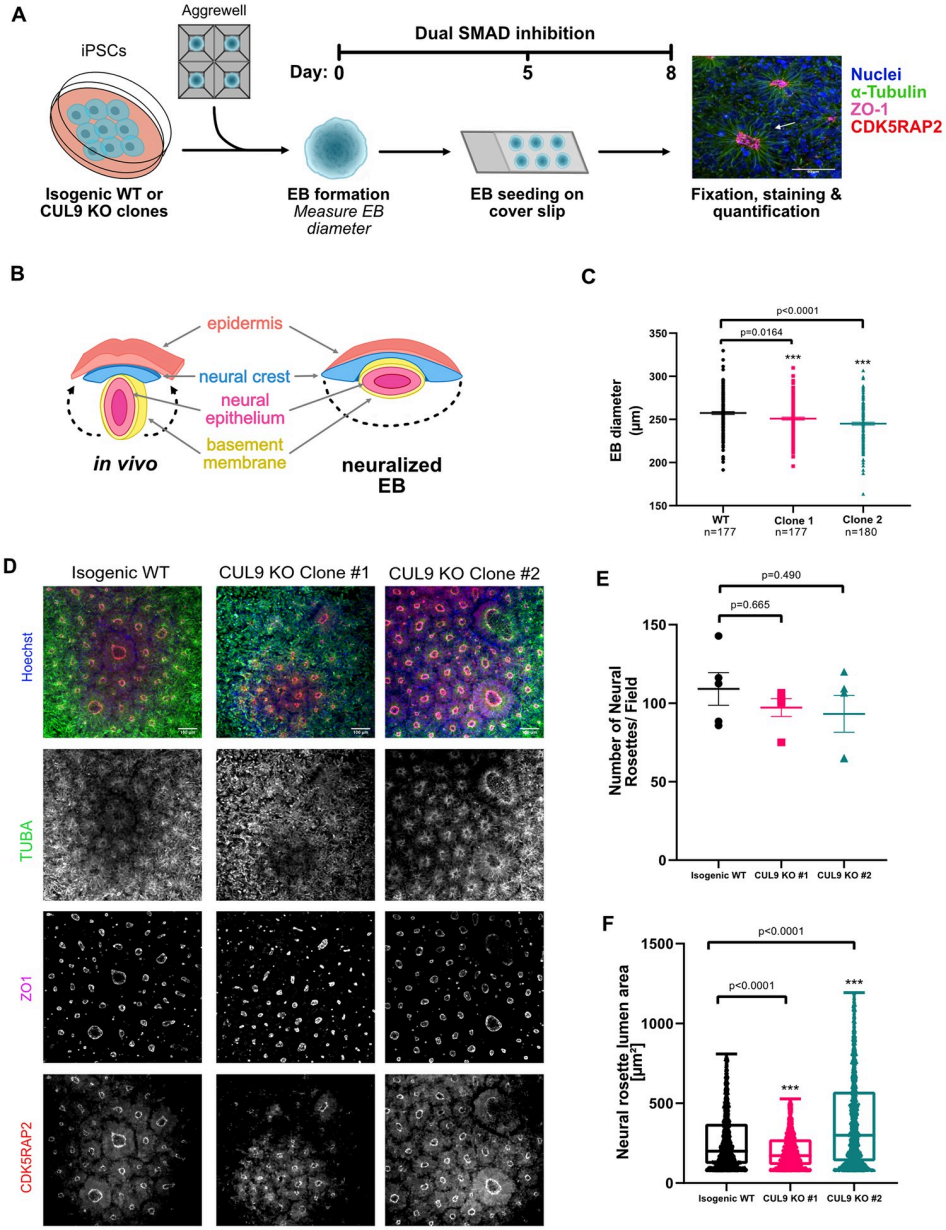
EBs and neural rosettes derived from CUL9 KO clones display abnormalities. (**A**) Protocol for EB and neural rosette differentiation. (**B**) Comparison of *in vivo* neural tube and *in vitro* neural rosette structure. Adapted from Haremaki et al., 2019 (**C**) The diameter of EBs derived from isogenic WT and CUL9 KO hPSCs derived EBs were imaged using an EVOS Inverted Fluorescent Microscope and the diameter of EBs was quantified using ImageJ. Mean and SEM were quantified. n = 3, number of EBs quantified in each biological replicated shown. (**D**) Isogenic WT and CUL9 KO EBs derived from hPSCs were differentiated by dual SMAD inhibition. Cells were fixed on day 8 of differentiation and stained for CDK5RAP2 (red), ZO1 (magenta), alpha-tubulin (TUBA, green) and Hoechst (blue). Scale bar = 100 μm. 10X objective. (**E**) Graph representing the average number of neural rosettes (NR) formed from a single EB (assumed to be a single field of view). 5 ROI per sample. Mean +/- SEM. P-value determined by one-way ANOVA. n = 3. (**F**) Graph representing the average size lumens within NRs rosettes. Thresholding ZO1 staining was used to count number of objects given set parameters. Area of ZO1 was calculated for each object, all lumenal areas per biological replicate were included individually in graph. 5 ROI per sample. P-values determined by one-way ANOVA; outliers removed using ROUT method. Median with min to max displayed. n = 3.

Neural rosettes serve as an *in vitro* model for neurulation, the early stages of neural tube formation [27, 28] (Fig 3A and 3B). Neural rosettes have a distinctive apical-basal polarity and can be easily identified by marker localization. ZO-1 (tight junction marker) and CDK5RAP2 (centrosome marker) localize apically, marking the central lumen. Cells extend radially from the lumen, as demonstrated by TUBA (alpha tubulin) staining (Fig 3A). Previous data demonstrate that the cells surrounding the lumen have a similar expression profile to the cells within the neural epithelium layer of the neural tube [27, 29] (Fig 3B).

To determine the effect of CUL9 deletion on neural rosette formation, we first grew CUL9 KO clones in suspension with dual SMAD inhibitors to form EBs (Fig 3A). EB diameter was measured five days after initial seeding. EBs derived from CUL9 KO clones have a significantly reduced diameter (Fig 3C). Neural rosettes formed from these EBs have the expected morphology based on marker localization (Fig 3D). To quantify neural rosette morphology, ZO1 staining was used to quantify the number of rosettes formed per EB, as well as the area of the lumen. While we saw no significant differences in the number of neural rosettes per EB, we observed significant differences in lumen size in both clones despite clonal variability (Fig 3E and 3F). We corroborated this result in CUL9 KD rosettes as well. Both shRNAs used resulted in decreased lumen size (S7 Fig). Abnormal lumen size can be the result of asymmetric division of R-NPCs or dysregulation of apical membrane size which affects the apical-basal polarity of rosettes [27, 30].

### CUL9 interacts with several subunits of the APC/C

**Fig 4:**
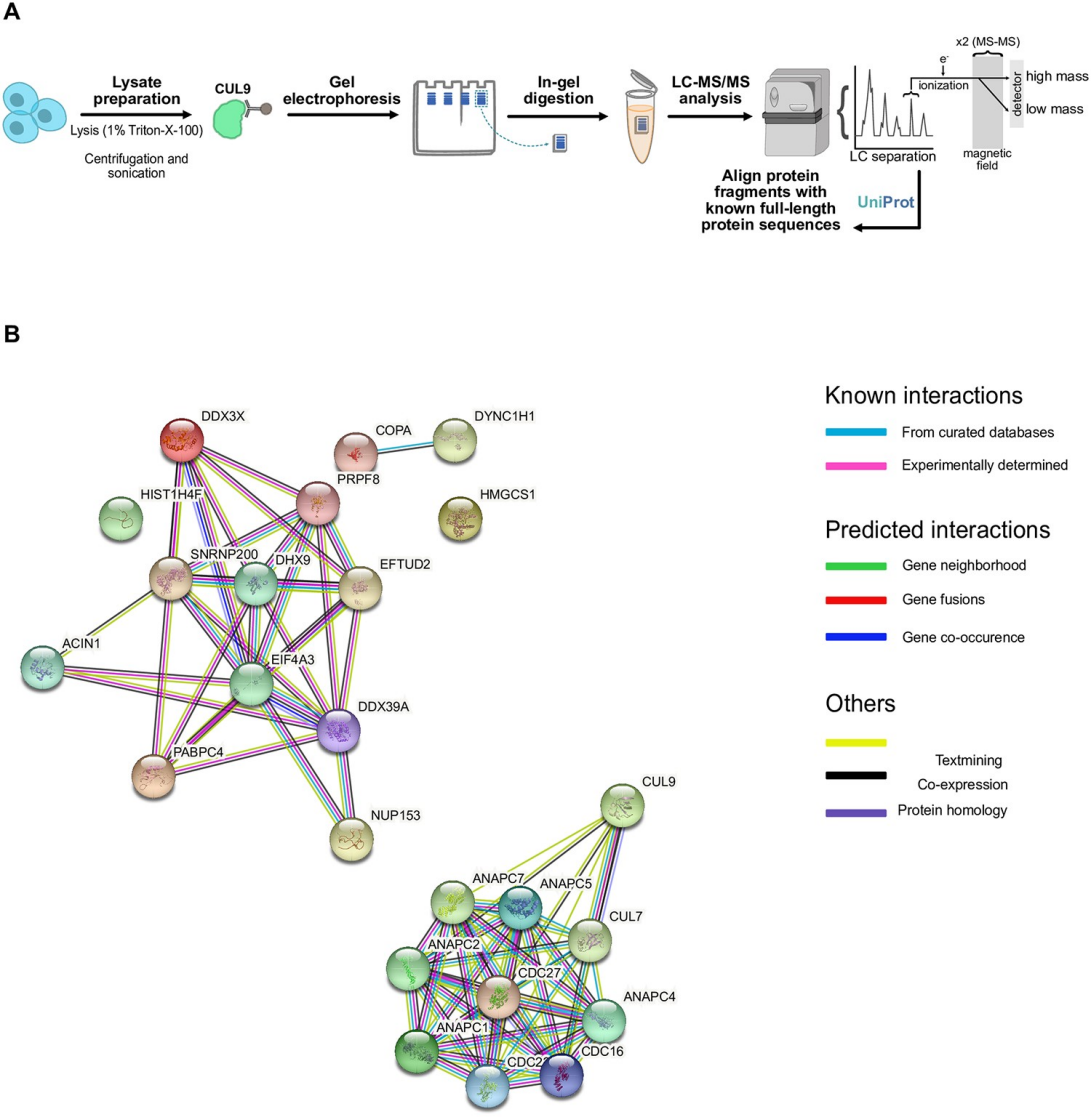
CUL9 interacts with several subunits of the APC/C. (**A**) Workflow for LC-MS/MS analysis of endogenous CUL9 immunoprecipitation from hPSC lysates. (**B**) Results from query using Search Tool for the retrieval of Interacting Genes/Proteins (STRING) [31–40]. Diagram depicts known and predicted direct and indirect protein-protein interactions amongst the significant proteins identified in the endogenous CUL9 immunoprecipitation from hESCs. Significance of proteins determined by Fisher’s exact test analyzed in Scaffold. Colored nodes represent queried proteins and the first shell of interactors. Clear nodes indicate second shell of interactors. Filled nodes show known, partial, or predicted structure. Empty nodes indicate structure information is not available for the given protein. All hits were statistically significant in one replicate (Fisher’s exact test, α = 0.05) and validated in at least three replicates.

To identify a potential CUL9 function during neuronal differentiation, we set out to identify CUL9 interacting proteins in hPSCs and hNSCs. We performed liquid chromatography tandem mass spectrometry (LC-MS/MS) in cells treated with a proteasome inhibitor (bortezomib) to enrich for potential CUL9 substrates (Fig 4A; S8A Fig). We identified 24 proteins significantly (1% FDR; Fisher’s exact p-value of <0.05) enriched in the hPSC CUL9 immunoprecipitation with a protein probability of 95% or higher in our initial LC-MS/MS analysis (Table 1; S1 Table; S8B Fig). We analyzed the direct and indirect interactions amongst our proteins of interest using the Search Tool for the Retrieval of Interacting Genes/Proteins (STRING) [31–39]. STRING is a database containing know protein-protein interactions. These include direct binding as well as indirect interactions including shared functions or common signaling pathways. Analysis of our dataset showed a group of protein interactions involved in transcriptional regulation and cell cycle regulation (Fig 4B). All the interacting proteins involved in cell cycle regulation, barring CUL7, were subunits of the APC/C. These were the only identified proteins in the CUL9 immunoprecipitation from hNPC lysates (S2 Table). A majority of the most enriched subunits in both hPSCs and hNSCs contained the tetratricopeptide repeat (TPR) domain, the only domain shared by multiple subunits within the APC/C (Table 1; S2 Table; Fig 4B). Considering the enrichment of TPR lobe subcomplex proteins in our LC-MS/MS data, we focused our efforts on determining if CUL9 regulates the TPR-containing subunits. The most significantly and consistently enriched protein in all replicates of LC-MS/MS analysis was a component of the APC/C known as APC7 (Fig 4B; S8B Fig). Additionally, it is solvent exposed, making it available for CUL9 binding. We validated the CUL9-APC7 interaction by co-immunoprecipitation in hPSCs and hNSCs (S5A Fig).

**Table 1:** Proteins significantly enriched in endogenous CUL9 immunoprecipitation from hPSC lysate as determined by LC-MS/MS.

Accesion Number	Protein name	Total spectral counts IP:CUL9	Total spectral counts IgG	% Coverage IP:CUL9	% Coverage IgG	Fisher’s Exact Test (p-value)	# of replicates
O75643	U5 small nuclear ribonucleoprotein 200 kDa helicase	38	14	25	9.3	0.009	2
Q08211	ATP-dependent RNA helicase A	34	15	31	16	0.044	3
Q9UJX3	Anaphase-promoting complex subunit 7	40	0	60	0	<0.0001	4
Q8IWT3	Cullin-9	29	0	17	0	<0.0001	4
Q9H1A4	Anaphase-promoting complex subunit 1	35	0	21	0	<0.0001	3
Q6P2Q9	Pre-mRNA-processing-splicing factor 8	24	5	14	4	0.005	2
P62805	Histone H4	26	8	44	34	0.013	3
Q9UJX4	Anaphase-promoting complex subunit 5	28	0	42	0	<0.0001	2
P38919	Eukaryotic initiation factor 4A-III	21	1	39	1.9	<0.0001	3
P30260	Cell division cycle protein 27 homolog	19	0	40	0	<0.0001	3
P49790	Nuclear pore complex protein Nup153	17	0	18	0	<0.0001	3
Q13042	Cell division cycle protein 16 homolog	21	0	30	0	<0.0001	3
Q14204	Cytoplasmic dynein 1 heavy chain 1	14	3	4.9	1.1	0.024	2
Q15029	116 kDa U5 small nuclear ribonucleoprotein component	38	14	16	3	0.014	2
Q9UJX2	Cell division cycle protein 23 homolog	15	0	29	0	0.00019	3
Q9UJX5	Anaphase-promoting complex subunit 4	17	0	26	0	<0.0001	3
P53621	Coatomer subunit alpha						2
Q9UJX6	Anaphase-promoting complex subunit 2	8	0	14	0	0.01	3
Q9UKV3	Apoptotic chromatin condensation inducer in the nucleus	8	0	8.1	0	0.01	2
Q01581	Hydroxymethylglutaryl-CoA synthase, cytoplasmic	6	0	13	0	0.032	2
Q14999	Cullin-7	8	0	6	0	0.01	3
O00148	ATP-dependent RNA helicase DDX39A	15	0	41	0	<0.0001	2
Q13310	Polyadenylate-binding protein 4	7	0	15	0	0.018	2
O00571	ATP-dependent RNA helicase DDX3X	4	0	7.4	0	0.05	2

Protein significance was determined using a Fisher’s exact test, α < 0.05. All proteins were identified using a 95% protein probability or higher, a 1% FDR, and present in at least two hPSC LC-MS/MS replicates. Relevant LC-MS/MS information in table is from a representative LC-MS/MS run.

### CUL9 does not regulate APC/C levels

**Fig 5:**
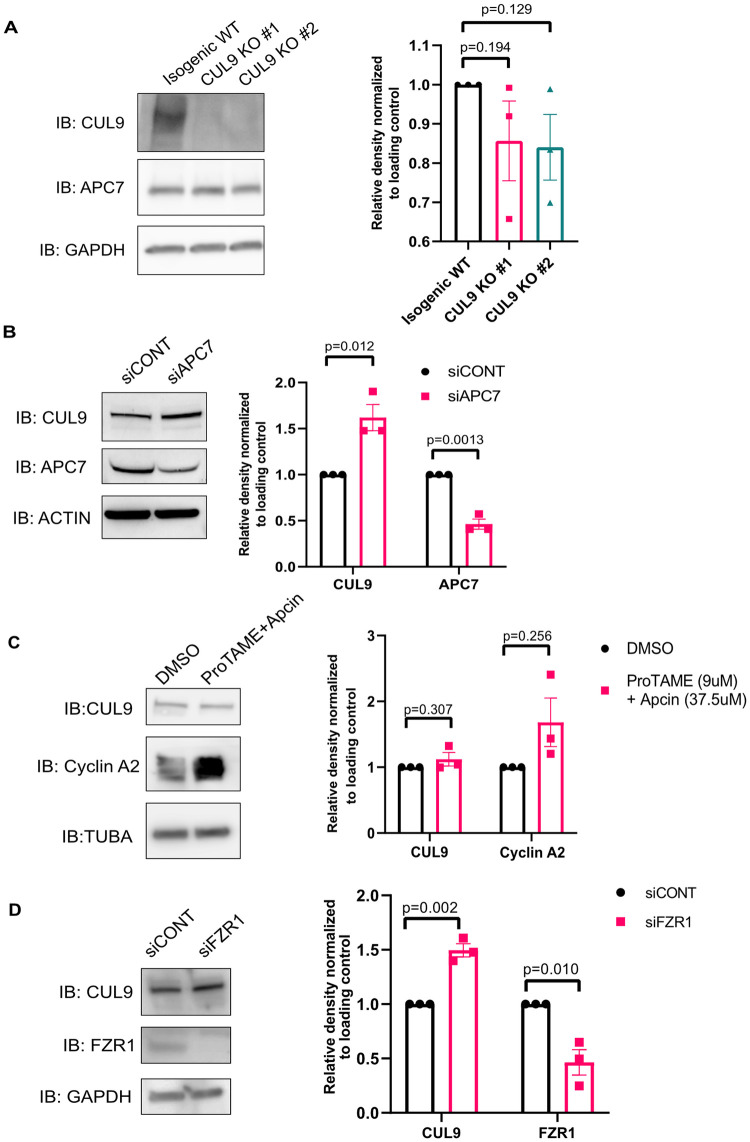
CUL9 protein levels are altered by depletion of APC/C subunits in hPSCs. (**A**) CUL9 KO clones have equivalent protein levels of APC7 to control cells as determined by Western blotting. n = 3; mean +/- SEM; Analysis done using student’s t-test, α = 0.05. Depletion of APC7 results in increased CUL9 levels in hPSCs (n = 3). (**B**) Downregulation of APC7 results in significantly increased CUL9 levels as determined by Western blotting. mean +/- SEM; Analysis done using student’s t-test, α = 0.05. (**C**) Chemical inhibition of APC/C-CDC20 does not affect CUL9 protein levels. Cells were treated with either DMSO or both 9uM ProTAME (Tocris) and 37.5 uM Apcin (Tocris) for 24 hours. While APC/C-CDC20 substrate cyclin A2 is consistently increased in treated cells, CUL9 levels are unchanged. Analysis done using student’s t-test, α = 0.05 n = 3. (**D**) siRNA mediated knockdown of FZR1 shows significant increase in CUL9 protein levels as determined by Western blotting. n = 3; mean +/- SEM; Analysis done using student’s t-test, α = 0.05.

**Fig 6:**
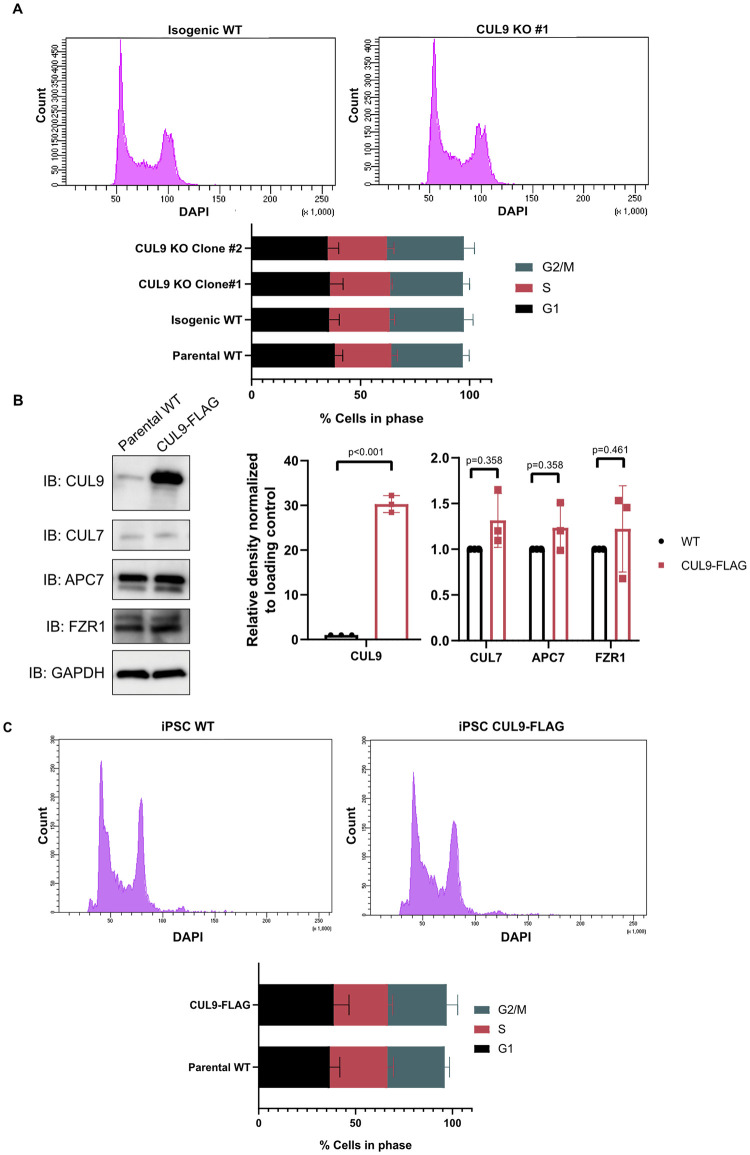
CUL9 does not regulate cell cycle progression in hPSCs. (**A**) CUL9 KO cells have a normal cell cycle profile. DNA content analysis by flow cytometry was done on CUL9 KO cells and control cells. Cell phases were determined by DNA content. <50 A.U. Pacific Blue, or <1N = sub-g1; 50–60 A.U Pacific blue, 1N, G1; 60–90 A.U. Pacific blue, 1<N<2, S; 90–120 A.U. Pacific blue, 2N, G2/M; >120 A.U. Pacific blue, >2N, Polyploidy. n = 3; mean +/- SD. (**B**) Lentiviral stable CUL9-FLAG overexpression line shows 30-fold increase in CUL9 protein levels, but no changes in APC7 or FZR1 levels as determined by Western blotting. n = 3; mean +/- SEM; Analysis done using student’s t-test, α = 0.05. (**C**) CUL9-FLAG expressing cells have normal cell cycle profile. DNA content analysis by flow cytometry was done on CUL9-FLAG expressing cell line and control cell line. Cell phases were determined by DNA content as described in A. n = 3; mean +/- SD.

To determine if APC/C subunits were targeted by CUL9 for ubiquitin mediated degradation, we examined if APC7 protein levels were enriched in CUL9 KO cells. Protein levels of APC7 were not significantly changed in either CUL9 KO clone as determined by Western blotting (Fig 5A). While ubiquitination canonically promotes proteasomal degradation of substrates, it can also result in a change in protein-protein interactions or protein localization and function. Based on our results, we postulated that CUL9 ubiquitination or interaction with the APC/C may play a role outside of protein stability. To determine if CUL9 affected APC/C function in cell cycle, we determined the cell cycle profile of CUL9 KO cells using flow cytometry analysis of DNA content. However, we did not see significant differences between control cells and CUL9 KO cells (Fig 6A).

### CUL9 protein levels increase when TPR subunit APC7 or adapter protein FZR1 are depleted

Since CUL9 does not appear to regulate the APC/C, we tested if APC/C regulates CUL9 stability. We noticed a significant increase in CUL9 protein levels when APC7 was depleted (Fig 5B). Since previous research has reported that TPR domain subunits of the APC/C play an important role in recruitment of APC/C adapter proteins, we sought to determine if CUL9 protein levels were affected by inhibition of adapter protein recruitment [41]. APC/C substrate specificity is mediated by its association with two adapter proteins: CDC20 and FZR1 (also known as CDH1). The APC/C targets mitotic cyclins when associated with CDC20, promoting mitotic progression, and G1-cyclins when associated with FZR1, promoting G1 progression.

Both APC/C-FZR1 and CDC20 recognize substrates by specific degrons. One such degron is the D box motif. Canonical APC/C substrates like cyclin B and cyclin A contain D box motifs [42, 43]. We used the APC/C degron repository resource created through a collaboration between the Davey laboratory at The Institute of Cancer Research and the Morgan laboratory at the University of California, San Francisco to analyze the CUL9 protein sequence for potential APC/C degrons. The APC/C degron prediction tool employs ProViz to scan a protein of interest for regions like previously characterized degrons [44]. This resource identified a potential D-box motif (RxxL) (TTRTILMMLLNR, amino acids 912–923) with a high consensus similarity to known motifs (Similarity score = 0.83). This motif was also highly disordered (Disorder score = 0.47), a common feature of degrons [45, 46].

Since both APC/C complexes can recognize the D-box degron, we tested if inhibition of CDC20 or FZR1 association would affect CUL9 protein levels. We first investigated if inhibition of APC/C-CDC20 affected CUL9 protein levels. To inhibit APC/C-CDC20, we treated the cells with two chemical inhibitors: ProTAME (tosyl-L-arginine methyl ester) and apcin (APC/C inhibitor). ProTAME blocks the APC/C-CDC20 interaction, and apcin binds to CDC20 and competitively inhibits ubiquitination of APC/C-CDC20 specific substrates [47]. Although we saw a consistent increase in the APC/C-CDC20 substrate Cyclin A2, CUL9 protein levels remained the same (Fig 5C). However, when we depleted FZR1 protein levels using siRNA, we saw a significant increase in CUL9 protein levels (Fig 5D). Despite this, we did not see any change in cell cycle profile or expression of APC/C subunits and adapter proteins when CUL9 was overexpressed in hPSCs (Fig 6B and 6C).

### iTRAQ reveals that CUL9 deletion alters protein levels of metabolic proteins

**Fig 7:**
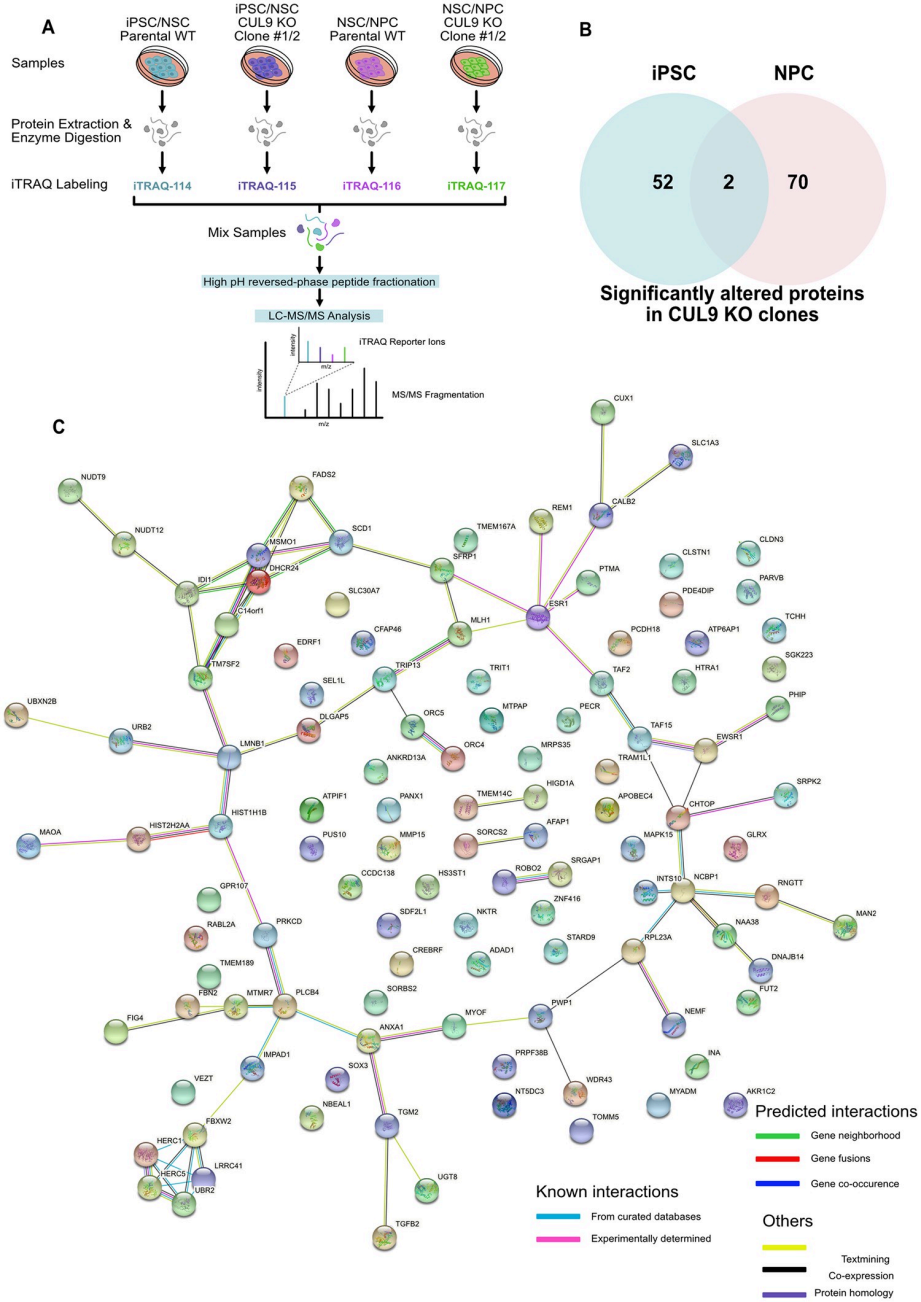
CUL9 KO cells have significantly altered levels of key proteins in metabolism as determined by iTRAQ. (**A**) Protocol for iTRAQ labeling of samples. Two comparisons were done: (1) iPSC WT, iPSC CUL9 KO, NSC WT, and NSC CUL9 KO. (2) NSC WT, NSC CUL9 KO, NPC WT, and NPC CUL9 KO. Each comparison was done for CUL9 KO #1 (n = 1) and #2 (n = 1). (**B**) Venn diagram showing number of significantly altered proteins in iPSC iTRAQ data and hNPC data. Two proteins were significantly altered in both datasets. (**C**) Results from query using Search Tool for the retrieval of Interacting Genes/Proteins (STRING). Diagram depicts known and predicted direct and indirect protein-protein interactions amongst the significant proteins identified in CUL9 KO hPSCs and NPCs. Proteins displayed are significantly (B-H method) altered in one clone and have an average normalized fold change of ≥1.2 or ≤ 0.8 in the other. Colored nodes represent queried proteins and the first shell of interactors. Clear nodes indicate second shell of interactors. Filled nodes show known, partial, or predicted structure. Empty nodes indicate non known or predicted structure is available for the given protein.

Quantitative proteomic approaches have emerged as an innovative way to quantify dynamic changes in protein levels and protein interactions during differentiation [48–50]. To provide insight into CUL9 function during NSC differentiation, we performed an in-depth analysis of proteins significantly altered in CUL9 KO hPSCs and hNPCs compared to WT using Isobaric Tags for Relative and Absolute Quantification (iTRAQ). iTRAQ is an approach where protein isolated from as many as eight cell populations can be simultaneously analyzed using mass spectrometry by covalent labeling of peptides with stable isotope labeled molecules [51]. Using this method, we compared the proteomes of CUL9 KO cells and Parental WT cells (Fig 7A). Two four-plex iTRAQ experiments were performed: (1) iPSC WT, iPSC CUL9 KO, NSC WT, and NSC CUL9 KO. (2) NSC WT, NSC CUL9 KO, NPC WT, and NPC CUL9 KO. Each four-plex was completed in both isogenic KO clones, acting as replicates. Briefly, each of the protein samples were labeled with a unique iTRAQ reagent of equivalent mass. The iTRAQ reagents labeled all primary amine groups of the peptides. Once each sample had been labeled with its unique iTRAQ label (i.e. 114, 115, 116, or 117), the four samples were mixed at a 1:1 ratio (200ug total protein of each sample). The sample mixture was analyzed by LC-MS/MS for protein identification and quantification (Fig 7A; S9 Fig). WT iPSC and WT NPCs were compared to WT NSC samples to validate induction of key genes for each cell type (Tables 2 and 3). 52 total proteins were significantly altered in iPSC CUL9 KO clones and 70 in NPC CUL9 KO clones (Fig 7B; S3–S6 Tables).

**Table 2:** Markers of differentiation are increased in NSCs and markers of pluripotency are decreased.

Accesion Number	Protein name	Fold change
P61601	Neurocalcin-delta (NCLAD)	2.337549
P08670	Vimentin (VIM)	2.208392
P48681	Nestin (NES)	1.416172
Q7L190	Developmental pluripotency-associated protein 4 (DPPA4)	0.514056
P16422	Epithelial cell adhesion molecule (ECAM)	0.197921

Average normalized fold change of Clone #1 and Clone #2 is shown for significantly altered hits in iPSC and NPC datasets. Listed proteins are significantly (B-H method) altered in one clone and have an average normalized fold change of ≥1.2 or ≤ 0.8.

**Table 3:** Markers of differentiation are increased in NPCs as determined by iTRAQ analysis.

Accesion Number	Protein name	Fold change
Q6PUV4	Complexin-2 (CPLX2)	14.86192
O43602	Neuronal migration protein doublecortin (DCX)	2.861057
Q13509	Tubulin beta-3 chain (TUBB3)	2.564271
Q16650	T-box brain protein 1 (TBR1)	2.306249
P11137	Microtubule-associated protein 2 (MAP2)	2.168276

Average normalized fold change of Clone #1 and Clone #2 is shown for significantly altered hits in iPSC and NPC datasets. Listed proteins are significantly (B-H method) altered in one clone and have an average normalized fold change of ≥1.2 or ≤ 0.8.

**Fig 8:**
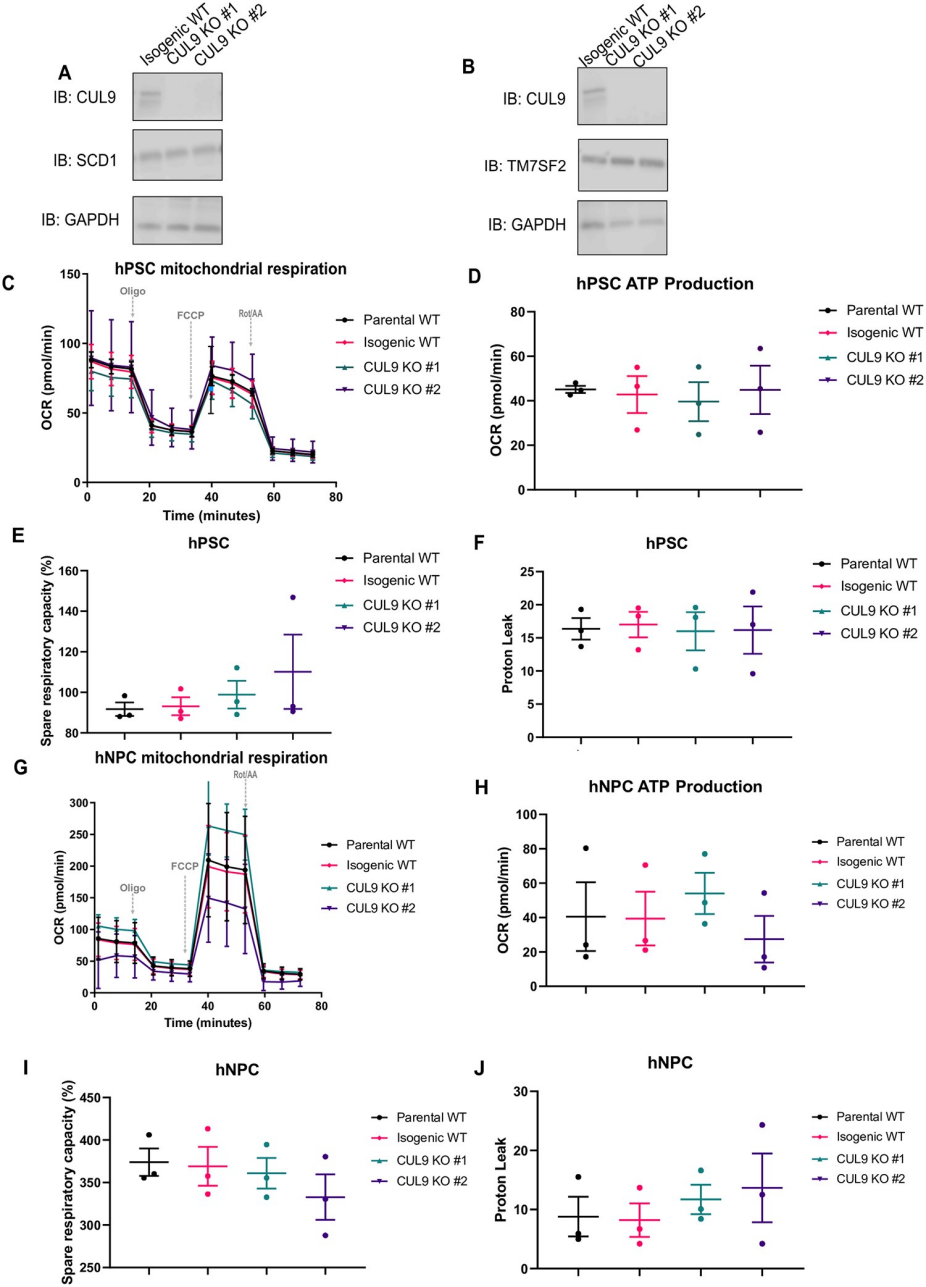
CUL9 KO hPSCs and hNPCs display normal metabolic profile. CUL9 KO express normal levels of key fatty acid metabolism enzymes SCD1 (**A**) and DHCR24 (**B**) n-3; mean +/- SEM; Analysis done using student’s t-test, α = 0.05. CUL9 KO hPSCs have the same oxygen consumption rate (OCR) (**C**), ATP production (**D**), spare respiratory capacity (**E**), and levels of proton leak (**F**) as parental and isogenic WT hPSCs. CUL9 KO hNPCs also display no abnormalities (**G-J**). n = 3 independent experiments done in triplicate, error bars are +/- SEM. (OCR) was measured using the Seahorse Biosciences Mito Stress Test on an XFe96 analyzer. ATP production was calculated from the corresponding OCR traces in panels A and C for each condition. Spare respiratory capacity is the difference between maximal respiration or basal respiration. Oligomycin inhibits ATP synthase interrupting the electron transport chain ultimately disrupting mitochondrial respiration and ATP production. FCCP is an uncoupler, uncoupling ATP production from the electron transport chain. Rotenone (complex I inhibitor) and antimycin A (complex III inhibitor) completely inhibit mitochondrial respiration, only permitting nonmitochondrial respiration to persist.

We searched our significantly altered (increased and decreased) hits against the STRING database and found two interesting clusters of interacting proteins: the HERC E3 ubiquitin ligases (bottom left) and a group of enzymes involved in fatty acid metabolism (upper left) (Fig 7). Additionally, we used the DAVID bioinformatics resource to identify trends in our iTRAQ data using gene ontology (GO) classification [52, 53]. Over 70% of significantly altered proteins were involved in metabolic processes as determined by GO analysis of biological processes (S10 Fig). Based on these analyses, we focused our efforts on characterizing the effects of CUL9 deletion and depletion on metabolic function. Most of the identified proteins are involved in fatty acid and steroid metabolic pathways. One of the significantly increased proteins, SCD1 (Acyl-coA desaturase) catalyzes the rate limiting reaction in the formation of monounsaturated fatty acids which are incorporated into key cellular structures like lipid membranes (S3 Table) [54, 55]. Another significantly increased protein, TM7SF2 (delta(14)-sterol reductase), is involved in a key reaction in cholesterol biosynthesis (S3 Table) [56]. However, there were no significant differences in protein levels of either proteins in CUL9 KO clones (Fig 8A) or CUL9 KD cells (S11A and S11B Fig). We also saw no changes in the oxygen consumption rate or ATP production in KO and KD hPSCs (Fig 8C–8F; S11C–S11F Fig) or hNPCs (Fig 8G–8J; S11G–S11J Fig).

### iTRAQ identifies altered levels of key neuronal transcription factors

GO analysis of the cellular compartment terms associated with the significantly altered proteins identified in CUL9 KO iPSCs and NPCs revealed that many of the proteins were localized to the nucleoplasm (Fig 6E). Two transcription factors stood out in this group of proteins: SOX3 and CUX1 (Homeobox protein cut-like 1) (S5 and S6 Tables). Both transcription factors coordinate neuronal gene expression to ensure the sequential differentiation of neuronal progeny during corticogenesis. Dysregulation in the early phases of corticogenesis can lead to significant neurodevelopmental abnormalities. Maintenance of the NPC pool in early cortical development is critical for appropriate production of cortical neurons [57, 58]. Overexpression of a single transcription factor can break this finely tuned process. For example, mice overexpressing PAX6 exhibit increased neurogenesis and a reduced number of neurons in the superficial layers of the cortex due to premature exhaustion of the NPC pool [59, 60]. Fine-tuning the expression levels of key transcription factors is key to normal cortical development.

We previously observed that both CUL9 KO clones have decreased levels of TUBB3 at the hNPC stage (S6A Fig). As TUBB3 expression is critical for neuronal maturation and synapse formation [21], we suspected a neuronal transcription factor upstream of TUBB3 may be involved in the observed abnormalities during neural rosette formation. iTRAQ revealed that the transcription factors SRY-box transcription factor 3 (SOX3) and Homeobox cut-like 1 (CUX1) were significantly increased in CUL9 KO hNPCs (S5 Table). SOX3 helps coordinate the transcriptional programs of precursors during early neurodevelopment and CUX1 transcriptional activity regulates neuronal maturation processes such as dendritic branching and synapse formation [61–64]. We did not observe altered levels of either protein by Western blotting (S12 Fig). However, we did observe variable expression of these proteins between biological replicates (S12 Fig).

## Discussion

Previous studies demonstrated that CUL9 promotes survival in sympathetic neurons and various cancer cells [9, 10]. Despite the prevalent role of most CRLs in development, mouse knockout models are viable and do not present a consistent phenotype [13]. In this study, we utilized a human cell-derived model to characterize CUL9 in human neuronal development. Our data indicate that CUL9 does not affect hPSC pluripotency or directly regulate apoptotic execution as previously described in other cell types. CUL9 mouse KO models have no apparent phenotypes in neuronal development [13, 14]. While we did not observe any affects in hPSCs or differentiated NSCs and NPCs, we did observe defects in neural rosette formation. This finding could indicate a distinction between CUL9 function in mouse and early human neurodevelopment.

Characterization of CUL9 in a human-derived cell system is critical, as CUL9 is a late evolutionary gene present in only vertebrates. We and others have hypothesized that CUL9 is a duplicate of the highly homologous CUL7, which is also present only in vertebrates [9, 14, 15]. This indicates that CUL9 may have a highly specified role in vertebrates, which may provide a plausible explanation for why CUL9 substrates have been so challenging to identify.

Our data showed CUL9 almost exclusively co-immunoprecipitated with APC/C subunits, specifically TPR subunits, in hPSCs and NSCs. The most enriched subunit in our analysis was APC7. Like CUL9, APC7 is also a late evolutionary gene that is only present in vertebrates. Its function has been similarly elusive, as APC7 KO cells are viable with minimal observed phenotypes [65]. Further characterization of the function of the CUL9-APC/C interaction could provide insight into the vertebrate specific functions of both proteins.

While APC7 KO cells do not show changes in cell cycle profile, they do show a slight decrease in ubiquitination of APC/C-FZR1 substrates [41, 65]. Depletion of APC7 and FZR1 both resulted in a small but significant increase in CUL9 protein levels. It is tempting to speculate that CUL9 is ubiquitinated by APC/C-FZR1 for proteasomal degradation, especially considering that CUL9 possesses a predicted APC/C degron, however, our methods do not directly test this hypothesis. An important first step would be validating the predicted D-box motif in CUL9. This could be done either by site directed mutations or in *vitro* experiments demonstrating direct binding and ubiquitination of CUL9 by APC/C-FZR1. The requirements for CUL9 activity and conformation are not known. For example, the APC/C has many phosphorylation sites required for its activation [66, 67]. Without an understanding of CUL9 structure and regulation by post-translational modifications such as NEDD8, *in vitro* studies and site directed mutations could be difficult to interpret. A basic understanding of the biochemical properties of CUL9 are needed to truly characterize its regulation or function.

The increase in CUL9 protein levels upon APC/C inactivation, though significant, are small. Additionally, increase or decrease in CUL9 protein levels do not affect cell cycle progression. We hypothesize that if APC/C-FZR1 targets CUL9 for proteasomal degradation it is likely specific to a phase of cell cycle and affects another process. This hypothesis is also supported by the CUL9-APC7 co-immunoprecipitation. Only a small amount of total endogenous CUL9 protein co-immunoprecipitates with APC7. If this interaction only occurs at a certain stage of cell cycle, not all cells in an asynchronous population would have the interaction at the time of sample collection. APC/C regulation is highly spatiotemporal, so a phase specific interaction is plausible. The APC/C has over 100 identified substrates and is predicted to have nearly double as many. These substrates are involved in processes ranging from apoptosis to metabolism. For example, APC/C-FZR1 is active during G2 phase where it regulates DNA damage response rather than cell cycle [68]. Future studies should address differential CUL9 regulation both throughout cell cycle and in different cell types.

CUL9 deletion does not affect cytosolic cytochrome *c* levels following induction of DNA damage, however, we do see less accumulation of cytochrome *c* when cells were treated with bortezomib. CUL9 may indirectly regulate apoptosis through regulation of proteasome independent pathways. Future studies could investigate if the CUL9-APC/C interaction is involved in apoptotic regulation or if apoptotic regulators identified by our iTRAQ or LC-MS/MS analysis like Apoptotic chromatin condensation inducer at the nucleus (ACIN1) (Table 1) are regulated by CUL9. These studies could reveal novel CUL9 functions in early differentiation and reveal new CUL9 regulated proteins.

Our quantitative proteomics data provide evidence that CUL9 function and regulation are likely dynamic during cellular differentiation. Over 100 proteins were significantly altered in iPSC and NPC CUL9 KO cells, but only two were significantly altered in both cell types. Overall, most of the combined hits were involved in lipid metabolism. 70% of the proteins classified in the oxidation reduction “metabolism” gene set were enriched in iPSCs, and most of these proteins were involved in fatty acid metabolism.

We sought to validate two proteins significantly increased in iPSCs, SCD1 and TM7SF2, involved in fatty acid desaturation and cholesterol biosynthesis. However, we did not detect a significant increase in these proteins by immunoblotting or detect any significant changes in oxygen consumption rate or ATP production. There were several other proteins involved in fatty acid synthesis we identified by iTRAQ like Fatty acid desaturase 2 (FADS2) and the delta-isomerase IDI1 that should be validated. Specific fatty acid metabolic assays using Seahorse analysis and metabolomics may also provide greater insight into any metabolic defects in CUL9 KO or KD cells.

The small changes we observe in CUL9 KO and KD proteins by iTRAQ could be because CUL9 regulates mRNA transcription through interaction or regulation with mRNA processing proteins rather than directly with the proteins identified by iTRAQ. For example, neuronal transcription factors CUX1 and SOX3 were significantly upregulated in CUL9 KO NPCs. However, this was not validated by immunoblotting, though, we did see great variation between biological replicates in both KO clones, indicating this is not just an effect of clonal variability. We also observed this variability when quantifying levels of pluripotency factors and neuronal markers. Interestingly, our LC-MS/MS analysis of CUL9 immunoprecipitation revealed CUL9 interaction with several proteins involved in mRNA processing and transcription like spliceosome component Small nuclear ribonucleoprotein U5 subunit 200 (SNRNP200) or RNA helicases DDX39A and DHX9. CUL9’s interactions with these proteins, direct or indirect, should be elucidated to explore if CUL9 plays a role in RNA processing. Regulation at the mRNA processing level could explain why CUL9 substrates remain elusive.

CUL9 interaction with other E3 ubiquitin ligases outside of the CUL7 also be investigated. CUL9 interaction with CUL1 and its F-Box associated protein FBXW2 should be explored, as FBXW2 was significantly increased in iPSCs–FBXW2 could even act as a substrate adapter for CUL9 as it does for CUL1 as no F-box proteins are currently known to associate with CUL9. Additionally, ubiquitin ligases outside of the CRL family may interact with CUL9. HERC5 was significantly increased in CUL9 KO hPSCS and HERC1 was significantly decreased in CUL9 KO hNPCs. Compensation by these E3 ubiquitin ligases should also be considered. Depletion and deletion of ligases often results in compensation by another. Compensation by one of these ligases could also explain why phenotypes have been absent or subtle in mouse and cellular models.

Future studies should investigate the role of CUL9 during the dynamic state of cell differentiation. If CUL9 is regulating transcription through mRNA processing, its role in cell fate transitions may be more conserved than previously appreciated. A more sensitive proteomics approach focused on screening differences in ubiquitinated protein populations rather than protein levels would allow identification of substrates ubiquitinated for purposes other than proteasomal degradation. A di-gly quantitative proteomics approach would allow for unbiased quantification of differentially ubiquitinated proteins in CUL9 KO cells [69]. Our study identifies CUL9 related pathways, but further analysis of CUL9 ubiquitination, function, and regulation during a variety cell transitions is required to understand where CUL9 fits into the crosstalk between these pathways and its contribution to early neurodevelopment.

## Materials and methods

### Cell culture

Human embryonic stem cell lines H9 (WA09) were obtained from WiCell Research Institute (Wisconsin). Human induced pluripotent stem cell line (GM25256) was obtained from Coriell. Cells were seeded as undifferentiated colonies on plates coated with Matrigel (Corning), maintained at 37°C and 5% CO2, and passaged as needed using Gentle Cell Dissociation Reagent (Stem Cell technologies). H9s were fed daily with mTeSR (Stem Cell Technologies), and iPSCs were fed daily with E8 (see S1 File for E8 preparation). Methods for neuronal and neural rosette differentiation included in S1 File.

### Cell treatments

Cell treatments were performed on 2–3 day old hPSC colonies. Cells were treated with proteasome inhibitor Bortezomib at a concentration of 0.5 μM. Pan-caspase inhibitor Q-VD-OPh (SM Biochemicals) was added to cells at a concentration of 25 μM. The DNA damaging agent etoposide was added to cells at a concentration of 1 μM of 20 μM for 3 or 24 hours. Cells were treated with both 9uM ProTAME (Tocris) and 37.5 μM Apcin (Tocris) for 24 hours. All stock solutions were prepared in DMSO and chemicals were added directly to the media alongside an equivalent DMSO only control.

### RNAi transfection

Commercially available siRNA (Thermo Fisher Scientific) was used to generate transient knockdowns of CUL9 and APC7 in hPSCs. hPSCs were seeded at 100,000 cells per well in a 6-well dish coated with Matrigel on d0. hNPCs were split 1:3 seven to nine days following neural induction on d0. Cells were transfected as per the manufacturer protocol using Lipofectamine RNAiMax (Thermo Fisher Scientific) in mTeSR on d1 and d2. Cells were left to recover for an additional 24 hours in fresh mTeSR or STEMDiff neural induction media, respectively. Cells were collected or fixed for analysis by Western blot on d4. Silencer Select Negative Control No. 1 (Thermo Fisher Scientific) was used as a control. The siRNA oligo sequences are as follows: CUL9 (s23061) 5’-GCUCGUCUACUUCACAAAtt-3’; APC7 (s229369) 5’-GCUGAACAGUAAUAGUGUUtt-3’; FZR1 (s27991) 5’-GGAUUAACGAGAAUGAGAAtt-3’; APC2 (s29882) 5’-CACUGGAUGUAUCUACAAtt-3’; APC10 (s20327) 5’-GAGCUCCAUUGGUAAAUUUtt-3’.

### CRISPR gene editing

hCUL9-/- GM25256 iPSCs were generated using CRISPR-Cas9. Briefly, a sgRNA was designed to target an early, conserved exon with at least 3bp of mismatch to any other site in the human genome to mitigate the risk of off-target editing (S1A Fig). GM25256 iPSCs were pretreated with mTeSR1 (Stem Cell Technologies) supplemented with 1X RevitaCell (Thermo Fisher Scientific) for 2 hours. Then, approximately 1X106 cells were transiently co-transfecting with precomplexed ribonuclear proteins (RNPs) consisting of 500 pmol of chemically modified sgRNA (hCUL9.sgRNA—5’- ugcucaugaccaagcacgag -3’, Synthego), 140 pmol of spCas9 protein (St. Jude Protein Production Core), and 500ng of pMaxGFP (Lonza). The transfection was performed via nucleofection (Lonza, 4D-Nucleofector™ X-unit) using solution P3 and program CA-137 in a large (100ul) cuvette according to the manufacturer’s recommended protocol. Cells were then plated onto Matrigel (Corning) coated plates into prewarmed (37C) mTeSR1 media supplemented with 1X RevitaCell. Several days post nucleofection, cells were single cell sorted on viability by flow cytometry and clonally selected as previously described [70]. Clones were screened and verified for the desired out-of-frame indel modifications via targeted deep sequencing using gene specific primers with partial Illumina adapter overhangs (hCUL9.F–5’- gacggtgccatggctgggaggtcag-3’ and hCUL9.R–5’-aatgtactcaccgccagcccagcct-3’, overhangs not shown) on a Miseq Illumina sequencer. NGS analysis of clones was performed using CRIS.py [71]. Normal karyotypes were validated using metaphase spread analysis (Genomic Associates) and pluripotency was validated using PluriTest microarray analysis (Thermo) [17].

### Western blotting

Cultured cells were lysed in 1% Triton buffer containing PMSF, PhosStop (Roche), and protease inhibitor cocktail. Protein concentrations were determined using the bicinchoninic acid method (Thermo Scientific), and 30–50 μg of protein was run on 4–20% Mini-Protean TGX precast protein gels (BioRad) in Tris-Gly-SDS buffer. Gels were then transferred onto polyvinylidene difluoride membranes at 4°C overnight to ensure sufficient transfer of large proteins. Membranes were blocked in 5% milk in 0.1% Tween prior to primary antibody incubation. We used antibodies against OCT4 (Cell Signaling Technology, Cat. 75463S), NANOG (Cell Signaling Technology, Cat. 4903S), SOX2 (Cell Signaling Technology, Cat. 5049S), Cleaved Caspase-3 (Cell Signaling Technology, Cat. 9661S), CUL9 (Bethyl Laboratories, Cat. A300-98A), CUL7 (Bethyl Laboratories, Cat. A300-223A), ANAPC7 (Bethyl Laboratories, Cat. A302-551), FZR1 (Abcam, Cat. ab3242), and Cyclin-A2 (Cell Signaling Technology, Cat. 4656T), PAX6 (Cell Signaling Technology Cat. 60433), Nestin (STEMCELL Technologies, Cat. 60091), TUBB3 (Cell Signaling Technology, Cat. 4466S), MAP2 (Thermo Fisher Scientific, Cat. 131500), beta-Actin (Sigma, Cat. A1978), alpha-Tubulin (Sigma, Cat. T9026), and GAPDH (Cell Signaling Technology, Cat. 5147S), SOX3 (Thermo Fisher, Cat. PA5-35983), TM7SF2 (Thermo Scientific, Cat. 12033-1-AP), CUX1 (Abcam, Cat. ab54583), and SCD1 (Bethyl, Laboratories, Cat. A305-259A-T). See full list of antibody information in S7 Table. HRP-conjugated secondary antibodies against mouse or rabbit IgG were purchased from Jackson Laboratories, and blots were developed with ECL Plus reagent (Amersham Biosciences). Clean-Blot IP detection reagent (HRP) (Life Technologies) was used in place of mouse or rabbit IgG secondaries for immunoprecipitation samples. Band density was determined using Image Studio Lite v5.2. Data was analyzed using student’s t-test.

### Immunofluorescence and microscopy

Cells were seeded on 35 mm glass bottom plates (Cellvis). Cells were fixed with 4% paraformaldehyde for 30 min at 4°C and permeabilized during blocking in 2% BSA containing 0.3% Triton X-100 for 1 hr at room temperature. After blocking, cells were treated with primary and secondary antibodies using standard methods. The following primary antibodies were used: OCT4 (Cell Signaling Technology, Cat. 75463S), NANOG (Cell Signaling Technology, Cat. 4903S), PAX6 (Cell Signaling Technology Cat. 60433), NESTIN (STEMCELL Technologies, Cat. 60091), TUBB3 (Cell Signaling Technology, Cat. 4466S), MAP2 (Thermo Fisher Scientific, Cat. 131500), Cytochrome *c* (BD Pharmingen, Cat. 556433). All secondary antibodies were conjugated to Alexa fluorophore derivatives (Thermo). See detailed information about antibodies in S7 Table. Nuclei were stained with Hoechst 3342 (Thermo). Fixed and stained cells were mounted with Fluoromount-G slide mounting medium (Electron Microscopy Sciences). Images were acquired using an Andor DU-897 camera mounted on a Nikon Spinning Disk. The software used for image acquisition and producing representative images was Nikon Elements.

### Real-time quantitative PCR

Total RNA was extracted using Tri Reagent Solution (Thermo). cDNA was prepared per the manufacturer’s protocol using the high capacity cDNA reverse transcription kit (Thermo). RT-qPCR was performed using a SYBR Green PCR Master Mix (Thermo) on a QuantStudio 3 Real-Time PCR System (Thermo) using the manufacturer provided standard curve protocol. mRNA levels and fold change were calculated using the comparative Ct method [72]. Glucose-6-phosphate isomerase (GPI) was used as a loading control for calculation of relative mRNA levels. The following primer sets were used: GPI: FW: 5’- GTGTACCTTCTAGTCCCGCC -3’ RV: 5’-GGTCAAGCTGAAGTGGTTGAAGC-3’; CUL9: FW: 5’-GAAGAACTCATTCGACAGAGGC-3’ RV: 5’-CAGTTGGCGTAGACCTCAGG-3’; OCT4: FW: 5’-GGGCTCTCCCATGCATTCAAAC-3’ RV: 5’-CACCTTCCCTCCAACCAGTTGC-3’; NANOG: FW: 5’-TGGGATTTACAGGCGTGAGCCAC-3’ RV: 5’-AAGCAAAGCCTCCCAATCCCAAAC-3’; MAP2: FW: 5’-CTCAGCACCGCTAACAGAGGE-3’ RV: 5’-CATTGGCGCTTCGGACAAG-3’; TUBB3: FW: 5’-GGCCAAGGGTCACTACACG-3’ RV: 5’-GCAGTCGCAGTTTTCACACTC-3’; TBR1: FW: 5’-GCAGCAGCTACCCACATTCA-3’ RV: 5’-AGGTTGTCAGTGGTCGAGATA-3’; EMX2: FW: 5’-CGGCACTCAGCTACGCTAAC-3’ RV: 5’-CAAGTCCGGGTTGGAGTAGAC-3’ Efficiency of primer sets were between 90–110% as determined by standard curve.

### Seahorse Mito Stress Test

iPSCs were plated in E8 media on Matrigel-coated Seahorse XF96 V3 PS cell culture microplates 2 days before the assay at 8 x 104 cells per well. One hour prior to the assay, media was switched to XF DMEM media containing 1 mM pyruvate, 2 mM glutamine, and 10 mM glucose. Oxygen consumption rate (OCR) was measured sequentially after addition of 1.0 μM oligomycin, 1.5 μM FCCP, and 0.5 μM rotenone/antimycin A. To obtain cortical neurons, iPSCs were differentiated and maintained as described in the Neuronal Differentiation section for 25 days [20]. Cortical neurons were plated in maintenance media on Matrigel-coated Seahorse XF96 V3 PS cell culture microplates 2 days before the assay at 8 x 104 cells per well. One hour prior to the assay, media was switched to XF DMEM media containing 1 mM pyruvate, 2 mM glutamine, and 10 mM glucose. Oxygen consumption rate (OCR) was measured sequentially after addition of 1.5 μM oligomycin, 1.5 μM FCCP, and 0.5 μM rotenone/antimycin A.

### Cell cycle analysis by flow cytometry

Cells were dissociated to a single cell concentration when they were 70–80% confluent using Gentle Cell Dissociation. Cells were washed 3X with PBS, then fixed in 70% EtOH at -20 °C for at least 2 hours. 1*106 cells were stained in 1mL of 1μg/mL of DAPI in a 0.1% Triton solution containing RNase overnight in the dark. Stained cells were analyzed using a 405nm (6-paramaters) laser of a 3-laser BD Fortessa. Forward and side scatter parameters were used to exclude doublets and debris. DAPI staining intensity was used to determine DNA content of cells. Gates for sub-G1, G1, S, G2/M, and polyploidy were determined based on DNA content.

### LC-MS/MS analysis of CUL9 immunoprecipitation

Immunoprecipitation experiments were performed by incubating 2mg of whole-cell lysates with 2 μg of PARC/H7AP1 (Bethyl, Cat. A300-98A), ANAPC7 (Bethyl, Cat. A302-551A) or FZR1 (Abcam, Cat. ab3242) for 1 hr at 4°C. 40 μL of 50/50 slurry of Dynabeads (Invitrogen) was added to sample and left to incubate for 1 hr at 4°C. Beads were separated by magnet and washed in 1% Triton. Protein was eluted and incubated with Elution Buffer (Invitrogen) and LDS/BME sample buffer at 95°C for 5 min. Immunoprecipitations were analyzed by Western blot or LC-MS/MS (See S1 File for detailed LC-MS/MS methods).

## Supporting information

S1 Fig Design and analysis of CUL9 KO hPSC clones. (**A**) Sequence of CUL9 exon 9 with antisense gRNA aligned to target sequence. Figure made using Benchling (**B**) CUL9 KO clones have single nucleotide insertions resulting in a frameshift mutation. Table summarizing the results of Next Generation Sequencing (NGS) analysis of control and CUL9 KO lines used for downstream experiments. Segment of CUL9 exon 9 sequenced for analysis by NGS are shown below table for each cell line. gRNA targeted sequence is highlighted in yellow, and single nucleotide insertions are highlighted in green. (**C**) CUL9 KO cells do not express CUL9 protein or increased levels of homologue CUL7. Western blot of analysis of control and CUL9 KO clones to analyze CUL9 and CUL7 protein levels. Isogenic WT and Clone #1 n = 4; Clone #2 n = 3; mean +/- SEM; Analysis done using student’s t-test, α = 0.05.
(TIF)

S2 Fig All cell lines used in this study have normal karyotypes. Metaphase spread of indicated cell line at indicated passage number displayed. Karyotype analysis was performed by Genomic Associates, Nashville, TN.
(TIF)

S3 Fig CUL9 KO clones have varied apoptotic resistance at exposure to low levels of DNA damaging agent etoposide. CUL9 KO cells and control cells were treated with 1 μM etoposide for 3 hours, and caspase 3/7 activity was measured using a CaspaseGlo assay. n = 5; +/- SEM; data analyzed using multiple t-tests, α = 0.05.
(TIF)

S4 Fig Deletion of CUL9 does not affect cytochrome *c* levels after its release from mitochondria during apoptosis. Parental WT (**A**) and CUL9 KO Clone #1 (**B**) were treated with the pan-caspase inhibitor Q-VD-OPh (25μM) and the DNA damaging agent etoposide (3 μM) or DMSO and collected for analysis at four hours after treatment. Clones were also treated with QVD, etoposide, and the proteasome inhibitor bortezomib (0.5 μM) Cells were stained with cytochrome *c* (cyt *c*) and Hoechst. In DMSO + Q-VD-Oph samples, cyt *c* is localized to the mitochondria. In cells treated with etoposide +QVD, cyt *c* is released from the mitochondria. When treated with bortezomib + etoposide +QVD, cytochrome *c* accumulates in the cytosol after it is released from the mitochondria. Boxed areas are enlarged below images, demonstrating the change in cyt *c* localization. Error bars = 100 μm.
(TIF)

S5 Fig CUL9 KO cells can differentiate to NSCs. **CUL9 KO NSCs were derived by standardized neuronal differentiation methods**. NSCs produced seven days after neuronal differentiation initiated. (**A**) The CUL9 and APC7 interaction was validated by co-immunoprecipitation in hESCs (n = 3) and hNSCs (n = 2). Input is 1.5% (30 μg) of total lysate used in immunoprecipitation (2mg). (**B**) CUL9 KO NSCs do not express CUL9 protein or increased levels of homolog CUL7. Neuronal differentiation of CUL9 KO hPSCs for seven days results in loss of pluripotency markers OCT4 and NANOG expression (**C**) as well as increased expression of NSC markers PAX6 and NESTIN (**D**). Isogenic WT and Clone #1 n = 4; Clone #2 n = 3; mean +/- SEM; Analysis done using student’s t-test, α = 0.05.
(TIF)

S6 Fig NPCs derived from CUL9 KO NSCs express key markers of neuronal differentiation. CUL9 KO NPCs were derived by standardized neuronal differentiation methods. NPCs were produced twenty-five days after neuronal differentiation initiated. (**A**) CUL9 KO NPCs do not express CUL9 protein or increased levels of homologue CUL7. Differentiation of hPSCs for 25 days results in increased expression of MAP2 and TUBB3; TUBB3 protein levels are significantly decreased in both clones as determined by Western blotting. Mean +/- SEM; Analysis done using student’s t-test, α = 0.05. n = 3. (**B**) Despite differences in TUBB3 at the protein level, RNA expression of B3TU (TUBB3) is unchanged. Analysis of RNA expression of markers EMX2, TBR1, and MAP2. RNA isolated from WT and CUL9 KO NPCs were analyzed by RT-qPCR. Error bars +/- SEM. iPSC. n = 3.
(TIF)

S7 Fig EBs and neural rosettes derived from CUL9 KD clones display abnormalities. (**A**) CUL9 KO cells express significantly decreased levels of CUL9 protein. Western blot of analysis of control and CUL9 KD clones to analyze CUL9 and CUL7 protein levels. n = 3; mean +/- SEM; Analysis done using student’s t-test, α = 0.05. (**B**) The diameter of EBs derived from isogenic shCONT and shCUL9 hPSC derived EBs were imaged using an EVOS Inverted Fluorescent Microscope and the diameter of EBs was quantified using ImageJ. Mean and SEM were quantified. n = 3, number of EBs quantified in each biological replicated shown. (**C**) shCONT and shCUL9 EBs derived from hPSCs were differentiated by dual SMAD inhibition. Cells were fixed on day 8 of differentiation and stained for CDK5RAP2 (red), ZO1 (magenta), alpha-tubulin (TUBA, green) and Hoechst (blue). Scale bar = 100 μm. 10X objective. (**D**) Graph representing the average number of neural rosettes (NR) formed from a single EB (assumed to be a single field of view). 5 ROI per sample. Mean +/- SEM. P-value determined by one-way ANOVA. n = 3. (**E**) Graph representing the average size lumens within NRs rosettes. Thresholding ZO1 staining was used to count number of objects given set parameters. Area of ZO1 was calculated for each object, all lumenal areas per biological replicate were included individually in graph. 5 ROI per sample. P-values determined by one-way ANOVA; outliers removed using ROUT. Median with min to max displayed. n = 3.
(TIF)

S8 Fig (A) Coomassie stained protein gel showing loading, IgG control, and CUL9 immunoprecipitation. All proteins from each lane were isolated from the gel. (B) Volcano plot showing significantly enriched hits in IgG control and CUL9 immunoprecipitation. Plot made using Scaffold. Analysis performed on Scaffold using Fisher’s exact test, p<0.05.
(TIF)

S9 Fig Representative histograms showing normalized distribution of (log) quantification protein ratios for (A) WT NSC vs. WT iPSC, (B) Clone #1 vs. WT iPSC, (C) WT NPC vs. WT NSC, and (D) Clone #1 NPC vs. WT NPC. (TIF)

S10 Fig Significantly enriched gene ontology (GO) terms obtained by DAVID functional annotation of significantly altered proteins. For each graph, only GO classifications that were at least 2-fold enriched in the respective data sets and for which at least two genes assigned to that term are represented. Significantly altered proteins were identified in Clone #1 or Clone #2 iPSC or hNPC datasets compared to WT iPSC or hNPC samples, respectively. Significance was determined by the Benjamini-Hochberg method. Proteins significantly (B-H method) altered in one clone with an average normalized fold change of ≥1.2 or ≤ 0.8 were included in GO analysis. (**A**) GO biological processes level one classifications enriched in full dataset. (**B**) Analysis of GO biological processes direct (BP) classifications enriched in proteins identified in “metabolism” level one classification shown in B (text highlighted in red in B). (**C**) GO cellular compartment direct (CC) classifications enriched in full dataset. GO classification terms highlighted in blue have corresponding tables listing classified proteins. (**D**) GO molecular function direct (MF) classifications enriched in full dataset.
(TIF)

S11 Fig CUL9 KO hNPCs have expected protein levels of key neuronal transcription factors. CUL9 KO cells express normal levels of transcription factors CUX1 and SOX3, n = 3, all independent, biological replicates shown in single blot to demonstrate variability between replicates.
(TIF)

S12 Fig CUL9 KD hPSCs and hNPCs have expected levels of key metabolic proteins and display normal metabolic profile. CUL9 KD cells express normal levels of key fatty acid metabolism enzymes SCD1 (**A**) and DHCR24 (**B**) n = 3; mean +/- SEM; Analysis done using student’s t-test, α = 0.05. CUL9 KO hPSCs have the same oxygen consumption rate (OCR) (**C**), ATP production (**D**), spare respiratory capacity (**E**), and levels of proton leak (**F**) as parental and shCONT hPSCs. CUL9 KD hNPCs also display no abnormalities (**G-J**). n = 3 independent experiments done in triplicate, error bars are +/- SEM. (OCR) was measured using the Seahorse Biosciences Mito Stress Test on an XFe96 analyzer. ATP production was calculated from the corresponding OCR traces in panels A and C for each condition. Spare respiratory capacity is the difference between maximal respiration or basal respiration. Oligomycin inhibits ATP synthase interrupting the electron transport chain ultimately disrupting mitochondrial respiration and ATP production. FCCP is an uncoupler, uncoupling ATP production from the electron transport chain. Rotenone (complex I inhibitor) and antimycin A (complex III inhibitor) completely inhibit mitochondrial respiration, only permitting nonmitochondrial respiration to persist.
(TIF)

S1 Table Proteins present in endogenous CUL9 immunoprecipitation from hPSC lysate, but not control IgG as determined by LC-MS/MS. All proteins were identified using a 95% protein probability or higher, a 1% FDR, and present in at least two hPSC LC-MS/MS replicates. Relevant LC-MS/MS information in table is from a representative LC-MS/MS run.
(XLSX)

S2 Table Proteins present in endogenous CUL9 immunoprecipitation from hNPC lysate as determined by LC-MS/MS. Identified proteins were present only in CUL9 immunoprecipitation and not in IgG control, with a 95% protein probability or higher, a 1% FDR, n = 1.
(XLSX)

S3 Table iTRAQ hits significantly upregulated in CUL9 KO hPSCs. Average normalized fold change of Clone #1 and Clone #2 is shown for significantly altered hits in iPSC and NPC datasets. Listed proteins are significantly (B-H method) altered in one clone and have an average normalized fold change of ≥1.2 or ≤ 0.8.
(XLSX)

S4 Table iTRAQ hits significantly downregulated in CUL9 KO hPSCs. Average normalized fold change of Clone #1 and Clone #2 is shown for significantly altered hits in iPSC and NPC datasets. Listed proteins are significantly (B-H method) altered in one clone and have an average normalized fold change of ≥1.2 or ≤ 0.8.
(XLSX)

S5 Table iTRAQ hits significantly upregulated in CUL9 KO NPCs. Average normalized fold change of Clone #1 and Clone #2 is shown for significantly altered hits in iPSC and NPC datasets. Listed proteins are significantly (B-H method) altered in one clone and have an average normalized fold change of ≥1.2 or ≤ 0.8.
(XLSX)

S6 Table iTRAQ hits significantly downregulated in CUL9 KO NPCs. Average normalized fold change of Clone #1 and Clone #2 is shown for significantly altered hits in iPSC and NPC datasets. Listed proteins are significantly (B-H method) altered in one clone and have an average normalized fold change of ≥1.2 or ≤ 0.8.
(XLSX)

S7 Table Detailed information about antibodies used in this study. (XLSX)

S1 File (DOCX)

S1 Raw images (PDF)
