# Supplementary figures and images for: A proteomics approach for the identification of cullin-9 (CUL9) related signaling pathways in induced pluripotent stem cell models
Source: PLoS One. 2021 Mar 11;16(3):e0248000. doi: 10.1371/journal.pone.0248000 (PMC7951927; doi:10.1371/journal.pone.0248000)

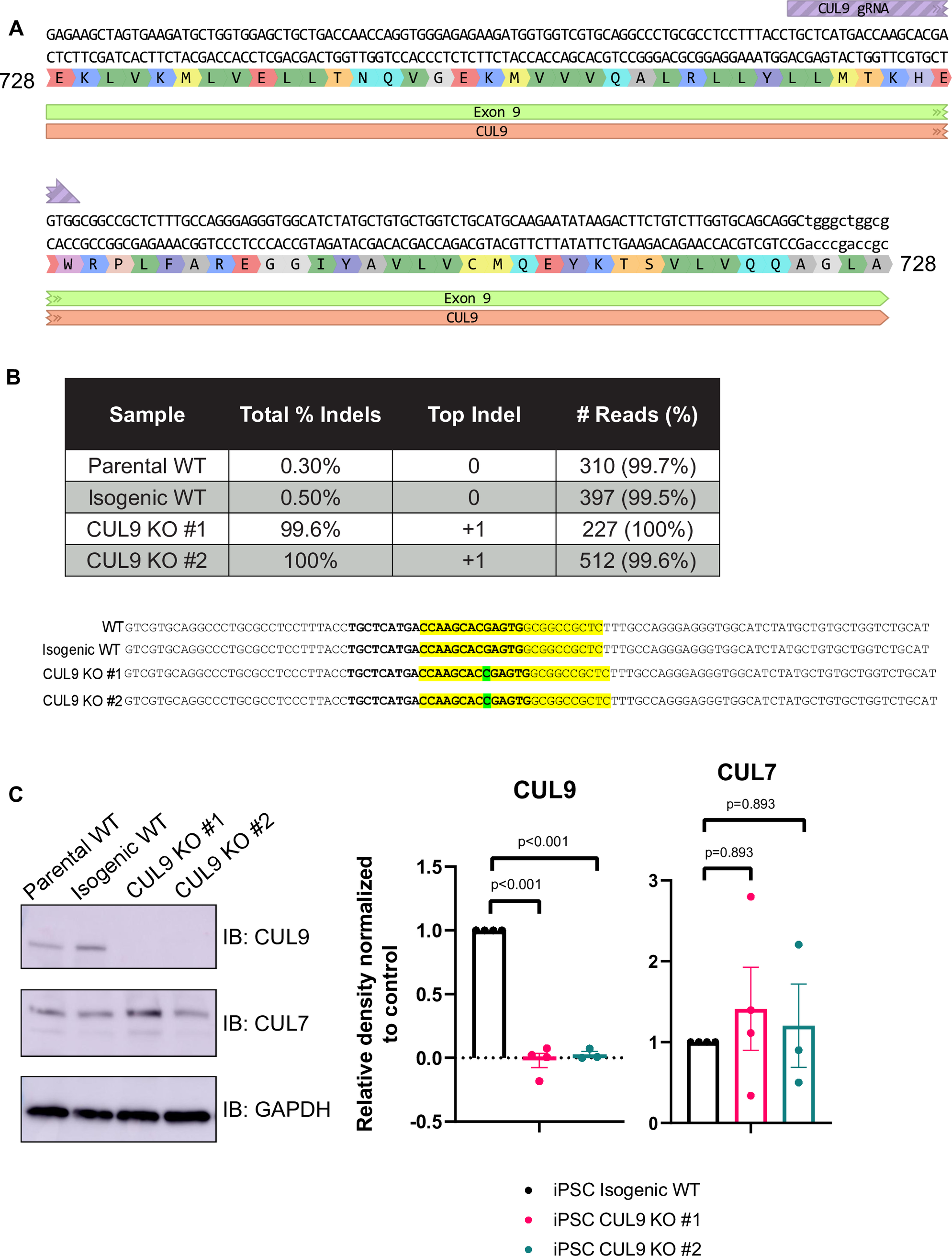

Supplement: S1 Fig — (A) Sequence of CUL9 exon 9 with antisense gRNA aligned to target sequence. Figure made using Benchling (B) CUL9 KO clones have single nucleotide insertions resulting in a frameshift mutation. Table summarizing the results of Next Generation Sequencing (NGS) analysis of control and CUL9 KO lines used for downstream experiments. Segment of CUL9 exon 9 sequenced for analysis by NGS are shown below table for each cell line. gRNA targeted sequence is highlighted in yellow, and single nucleotide insertions are highlighted in green. (C) CUL9 KO cells do not express CUL9 protein or increased levels of homologue CUL7. Western blot of analysis of control and CUL9 KO clones to analyze CUL9 and CUL7 protein levels. Isogenic WT and Clone #1 n = 4; Clone #2 n = 3; mean +/- SEM; Analysis done using student’s t-test, α = 0.05. (TIF) [file pone.0248000.s001.tif]

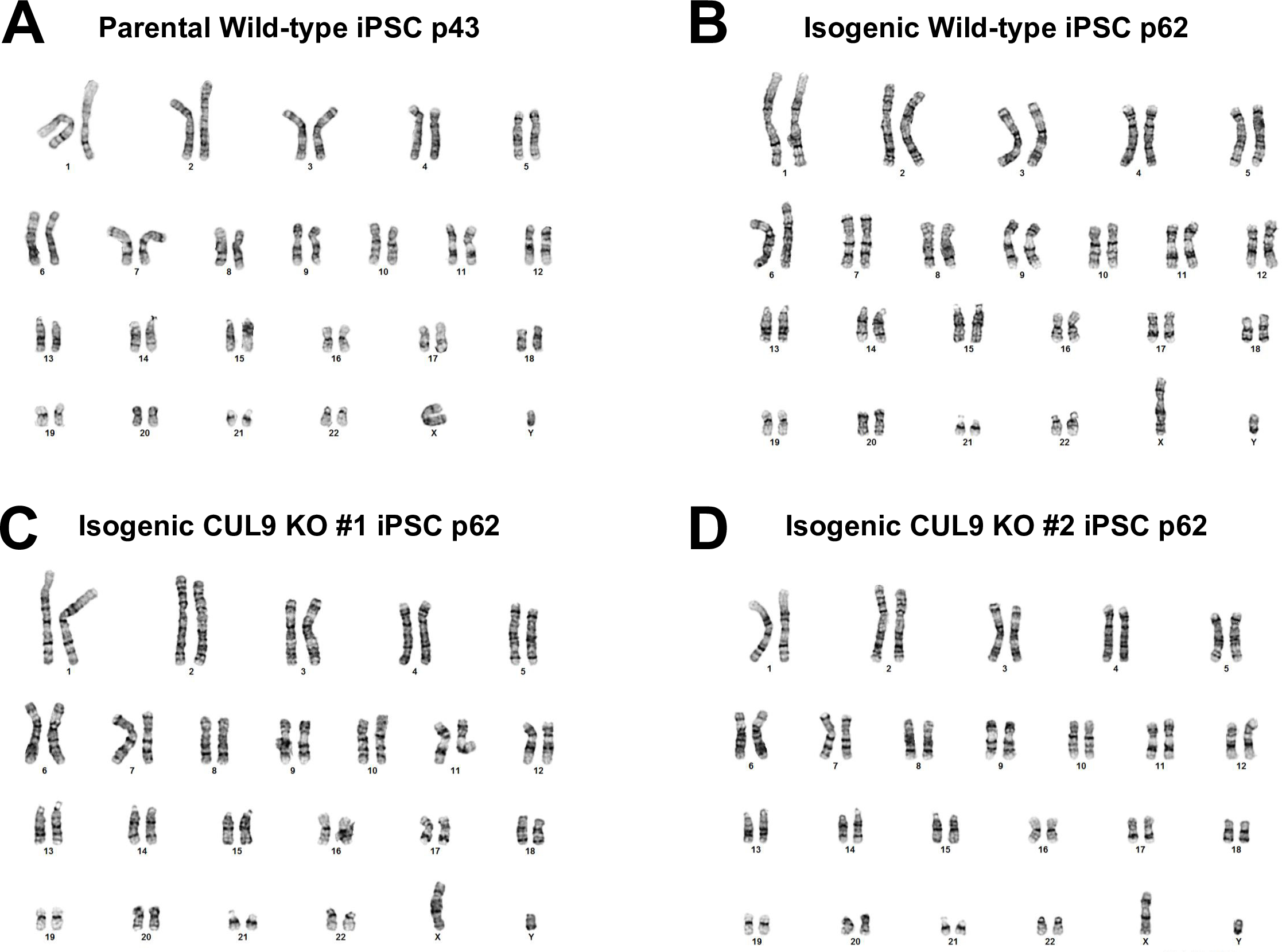

Supplement: S2 Fig — Metaphase spread of indicated cell line at indicated passage number displayed. Karyotype analysis was performed by Genomic Associates, Nashville, TN. (TIF) [file pone.0248000.s002.tif]

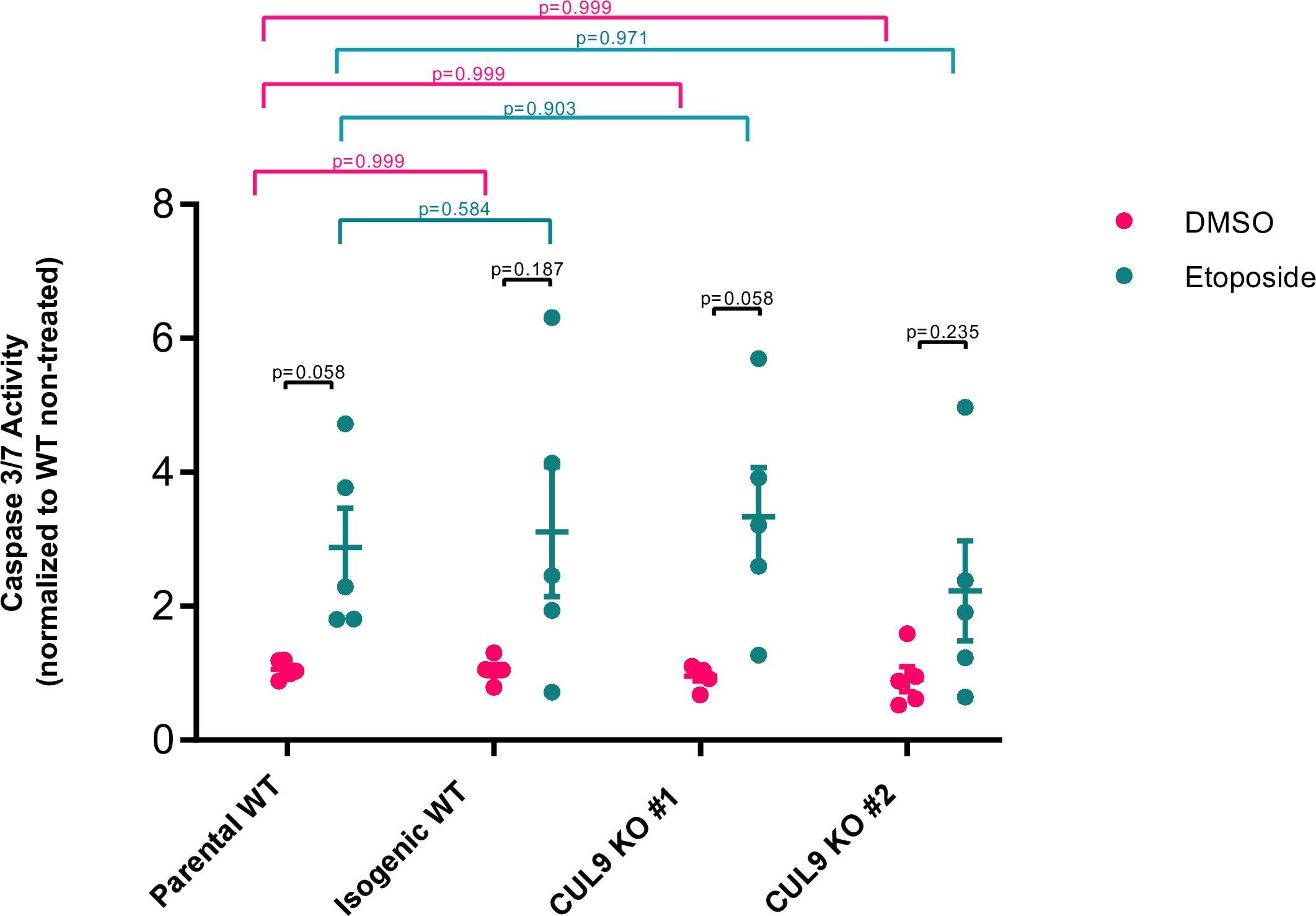

Supplement: S3 Fig — CUL9 KO cells and control cells were treated with 1 μM etoposide for 3 hours, and caspase 3/7 activity was measured using a CaspaseGlo assay. n = 5; +/- SEM; data analyzed using multiple t-tests, α = 0.05. (TIF) [file pone.0248000.s003.tif]

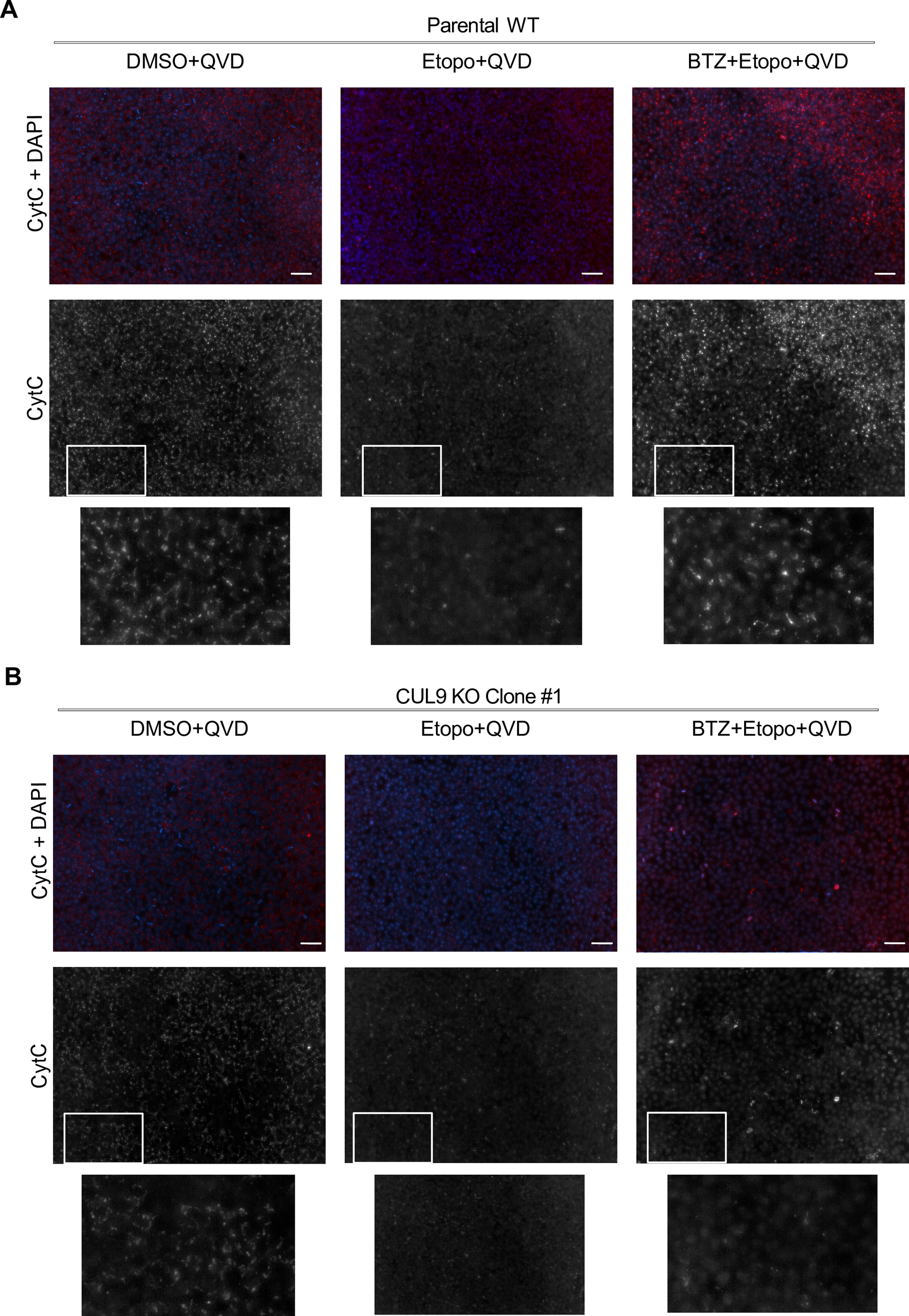

Supplement: S4 Fig — Parental WT (A) and CUL9 KO Clone #1 (B) were treated with the pan-caspase inhibitor Q-VD-OPh (25μM) and the DNA damaging agent etoposide (3 μM) or DMSO and collected for analysis at four hours after treatment. Clones were also treated with QVD, etoposide, and the proteasome inhibitor bortezomib (0.5 μM) Cells were stained with cytochrome c (cyt c) and Hoechst. In DMSO + Q-VD-Oph samples, cyt c is localized to the mitochondria. In cells treated with etoposide +QVD, cyt c is released from the mitochondria. When treated with bortezomib + etoposide +QVD, cytochrome c accumulates in the cytosol after it is released from the mitochondria. Boxed areas are enlarged below images, demonstrating the change in cyt c localization. Error bars = 100 μm. (TIF) [file pone.0248000.s004.tif]

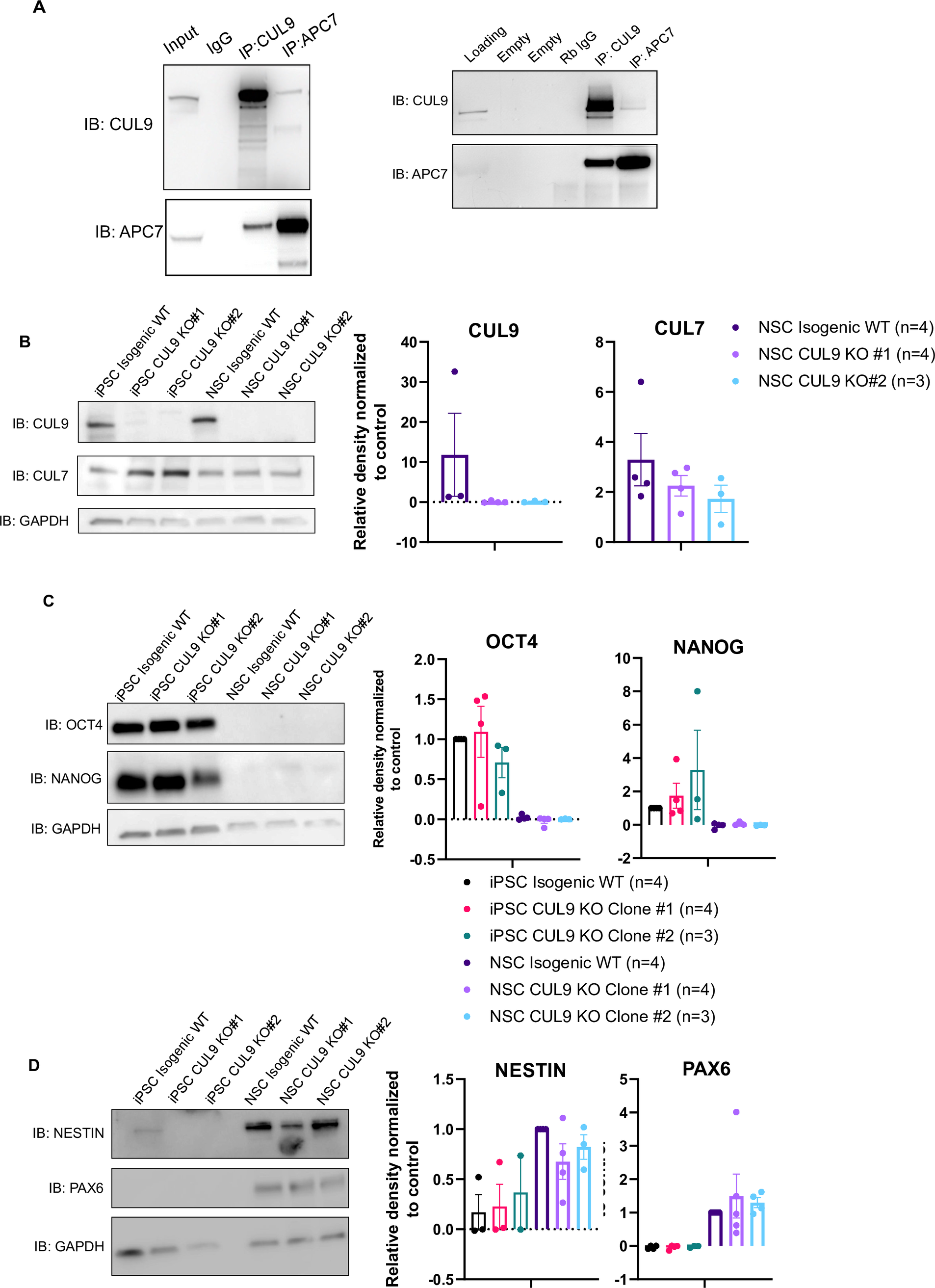

Supplement: S5 Fig — CUL9 KO NSCs were derived by standardized neuronal differentiation methods. NSCs produced seven days after neuronal differentiation initiated. (A) The CUL9 and APC7 interaction was validated by co-immunoprecipitation in hESCs (n = 3) and hNSCs (n = 2). Input is 1.5% (30 μg) of total lysate used in immunoprecipitation (2mg). (B) CUL9 KO NSCs do not express CUL9 protein or increased levels of homolog CUL7. Neuronal differentiation of CUL9 KO hPSCs for seven days results in loss of pluripotency markers OCT4 and NANOG expression (C) as well as increased expression of NSC markers PAX6 and NESTIN (D). Isogenic WT and Clone #1 n = 4; Clone #2 n = 3; mean +/- SEM; Analysis done using student’s t-test, α = 0.05. (TIF) [file pone.0248000.s005.tif]

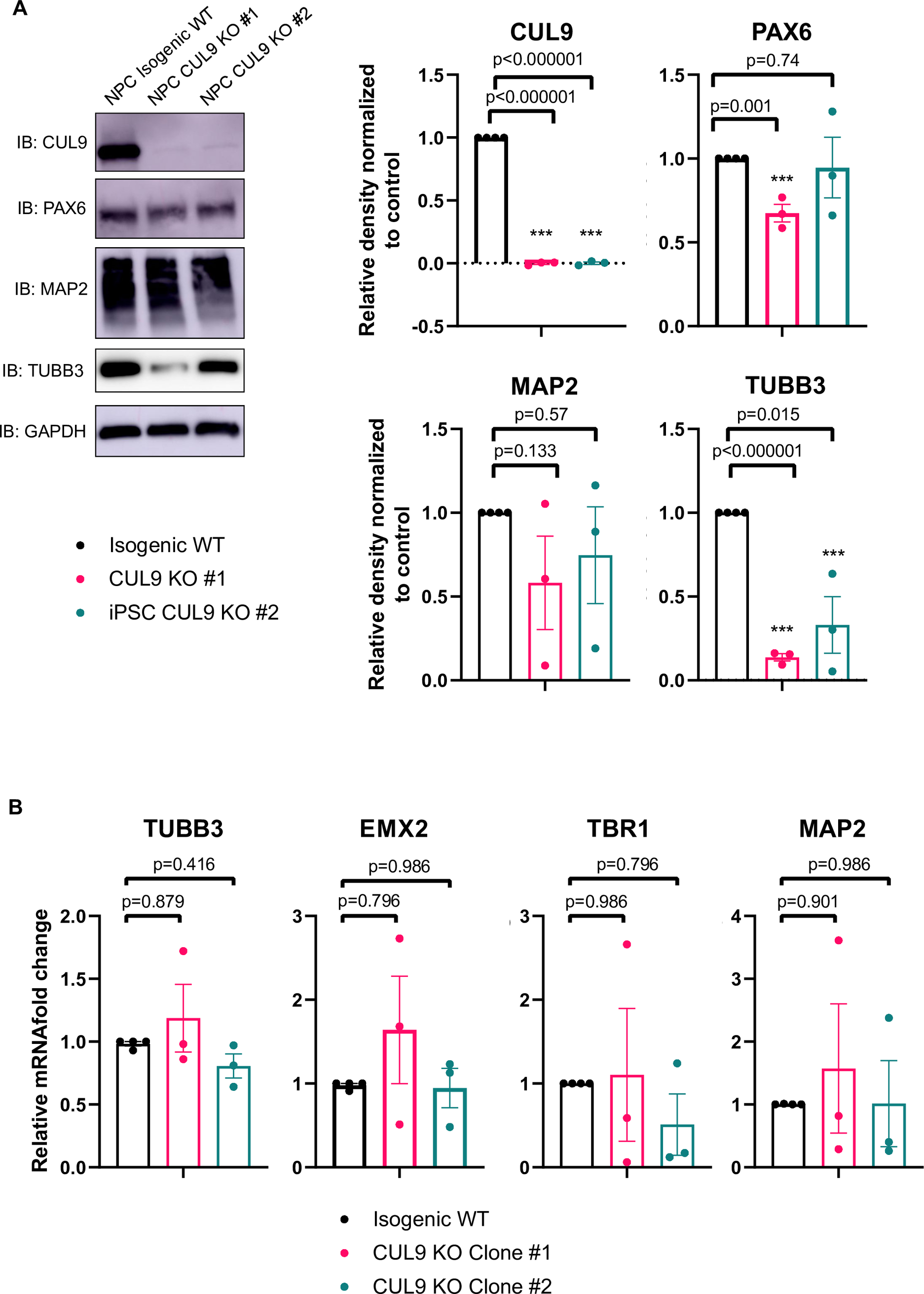

Supplement: S6 Fig — CUL9 KO NPCs were derived by standardized neuronal differentiation methods. NPCs were produced twenty-five days after neuronal differentiation initiated. (A) CUL9 KO NPCs do not express CUL9 protein or increased levels of homologue CUL7. Differentiation of hPSCs for 25 days results in increased expression of MAP2 and TUBB3; TUBB3 protein levels are significantly decreased in both clones as determined by Western blotting. Mean +/- SEM; Analysis done using student’s t-test, α = 0.05. n = 3. (B) Despite differences in TUBB3 at the protein level, RNA expression of B3TU (TUBB3) is unchanged. Analysis of RNA expression of markers EMX2, TBR1, and MAP2. RNA isolated from WT and CUL9 KO NPCs were analyzed by RT-qPCR. Error bars +/- SEM. iPSC. n = 3. (TIF) [file pone.0248000.s006.tif]

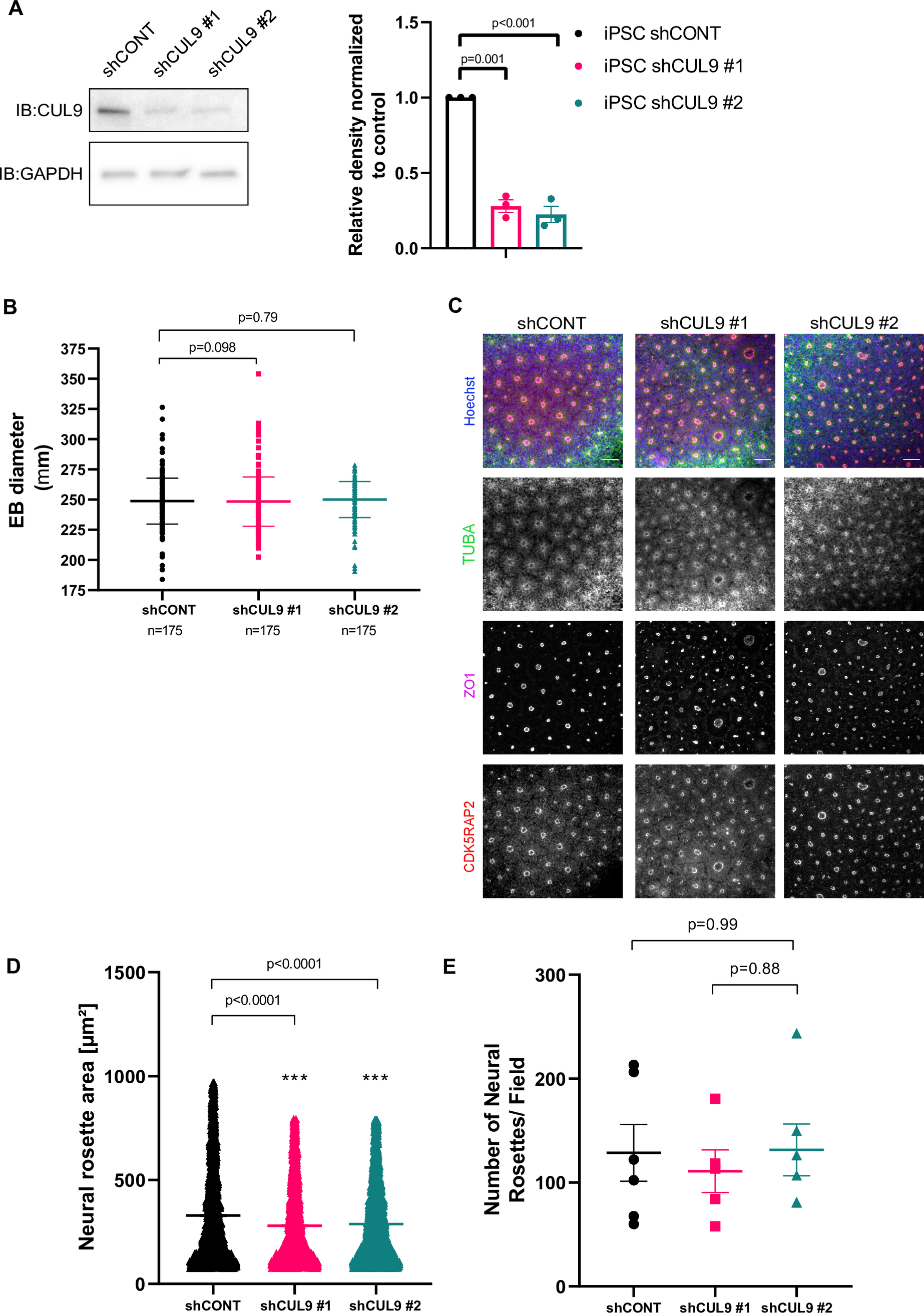

Supplement: S7 Fig — (A) CUL9 KO cells express significantly decreased levels of CUL9 protein. Western blot of analysis of control and CUL9 KD clones to analyze CUL9 and CUL7 protein levels. n = 3; mean +/- SEM; Analysis done using student’s t-test, α = 0.05. (B) The diameter of EBs derived from isogenic shCONT and shCUL9 hPSC derived EBs were imaged using an EVOS Inverted Fluorescent Microscope and the diameter of EBs was quantified using ImageJ. Mean and SEM were quantified. n = 3, number of EBs quantified in each biological replicated shown. (C) shCONT and shCUL9 EBs derived from hPSCs were differentiated by dual SMAD inhibition. Cells were fixed on day 8 of differentiation and stained for CDK5RAP2 (red), ZO1 (magenta), alpha-tubulin (TUBA, green) and Hoechst (blue). Scale bar = 100 μm. 10X objective. (D) Graph representing the average number of neural rosettes (NR) formed from a single EB (assumed to be a single field of view). 5 ROI per sample. Mean +/- SEM. P-value determined by one-way ANOVA. n = 3. (E) Graph representing the average size lumens within NRs rosettes. Thresholding ZO1 staining was used to count number of objects given set parameters. Area of ZO1 was calculated for each object, all lumenal areas per biological replicate were included individually in graph. 5 ROI per sample. P-values determined by one-way ANOVA; outliers removed using ROUT. Median with min to max displayed. n = 3. (TIF) [file pone.0248000.s007.tif]

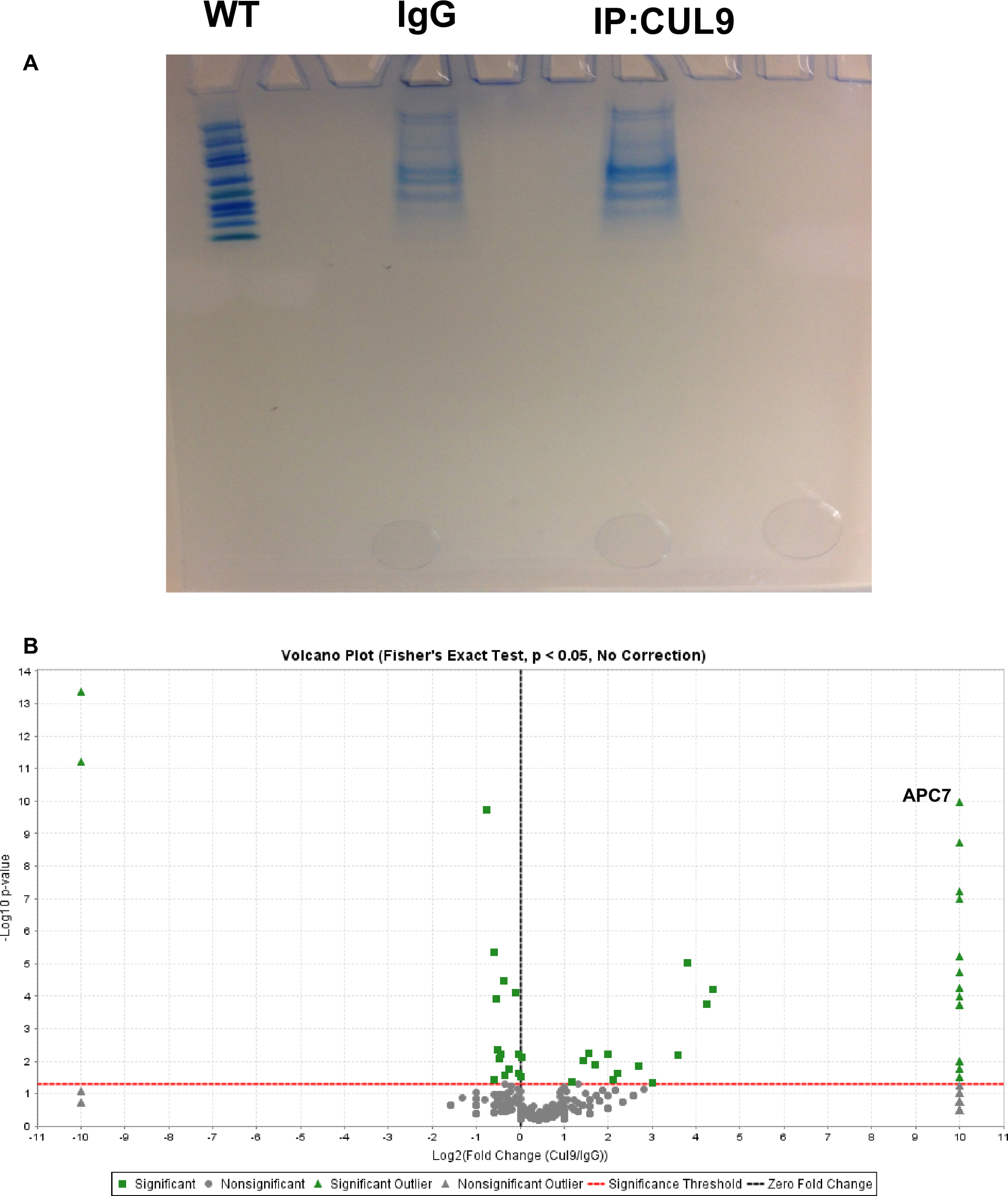

Supplement: S8 Fig — (A) Coomassie stained protein gel showing loading, IgG control, and CUL9 immunoprecipitation. All proteins from each lane were isolated from the gel. (B) Volcano plot showing significantly enriched hits in IgG control and CUL9 immunoprecipitation. Plot made using Scaffold. Analysis performed on Scaffold using Fisher’s exact test, p<0.05. (TIF) [file pone.0248000.s008.tif]

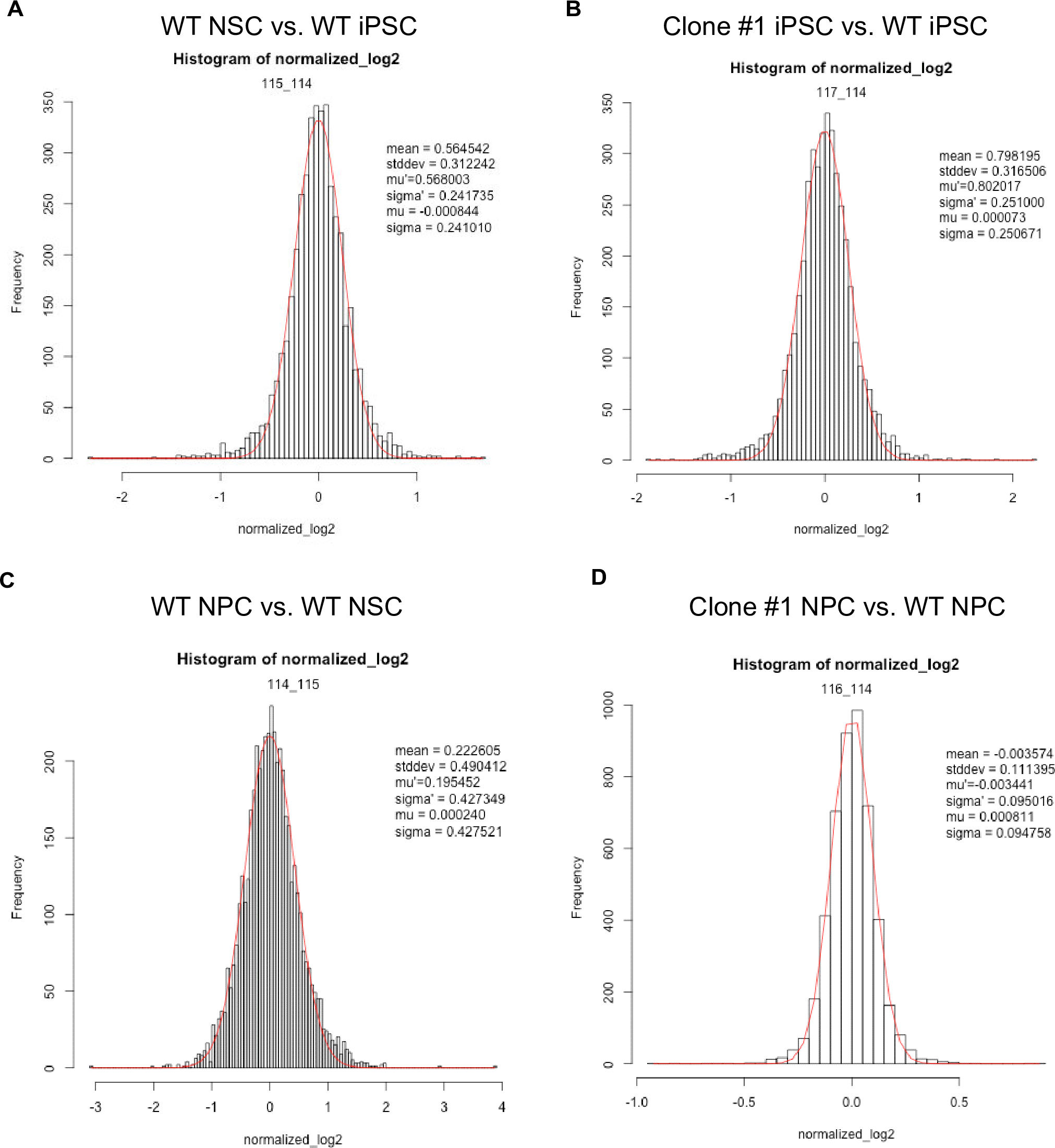

Supplement: S9 Fig — (TIF) [file pone.0248000.s009.tif]

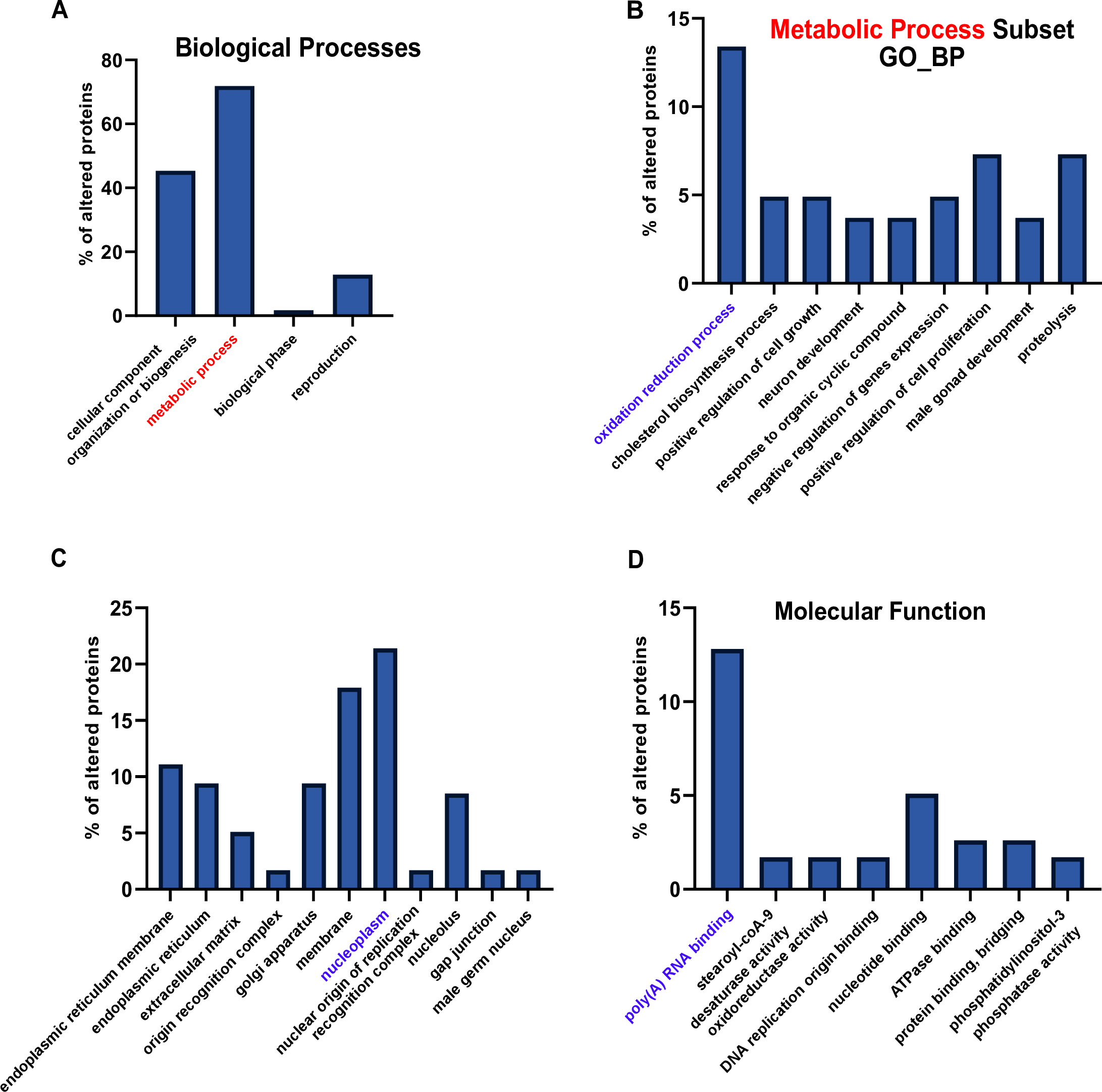

Supplement: S10 Fig — For each graph, only GO classifications that were at least 2-fold enriched in the respective data sets and for which at least two genes assigned to that term are represented. Significantly altered proteins were identified in Clone #1 or Clone #2 iPSC or hNPC datasets compared to WT iPSC or hNPC samples, respectively. Significance was determined by the Benjamini-Hochberg method. Proteins significantly (B-H method) altered in one clone with an average normalized fold change of ≥1.2 or ≤ 0.8 were included in GO analysis. (A) GO biological processes level one classifications enriched in full dataset. (B) Analysis of GO biological processes direct (BP) classifications enriched in proteins identified in “metabolism” level one classification shown in B (text highlighted in red in B). (C) GO cellular compartment direct (CC) classifications enriched in full dataset. GO classification terms highlighted in blue have corresponding tables listing classified proteins. (D) GO molecular function direct (MF) classifications enriched in full dataset. (TIF) [file pone.0248000.s010.tif]

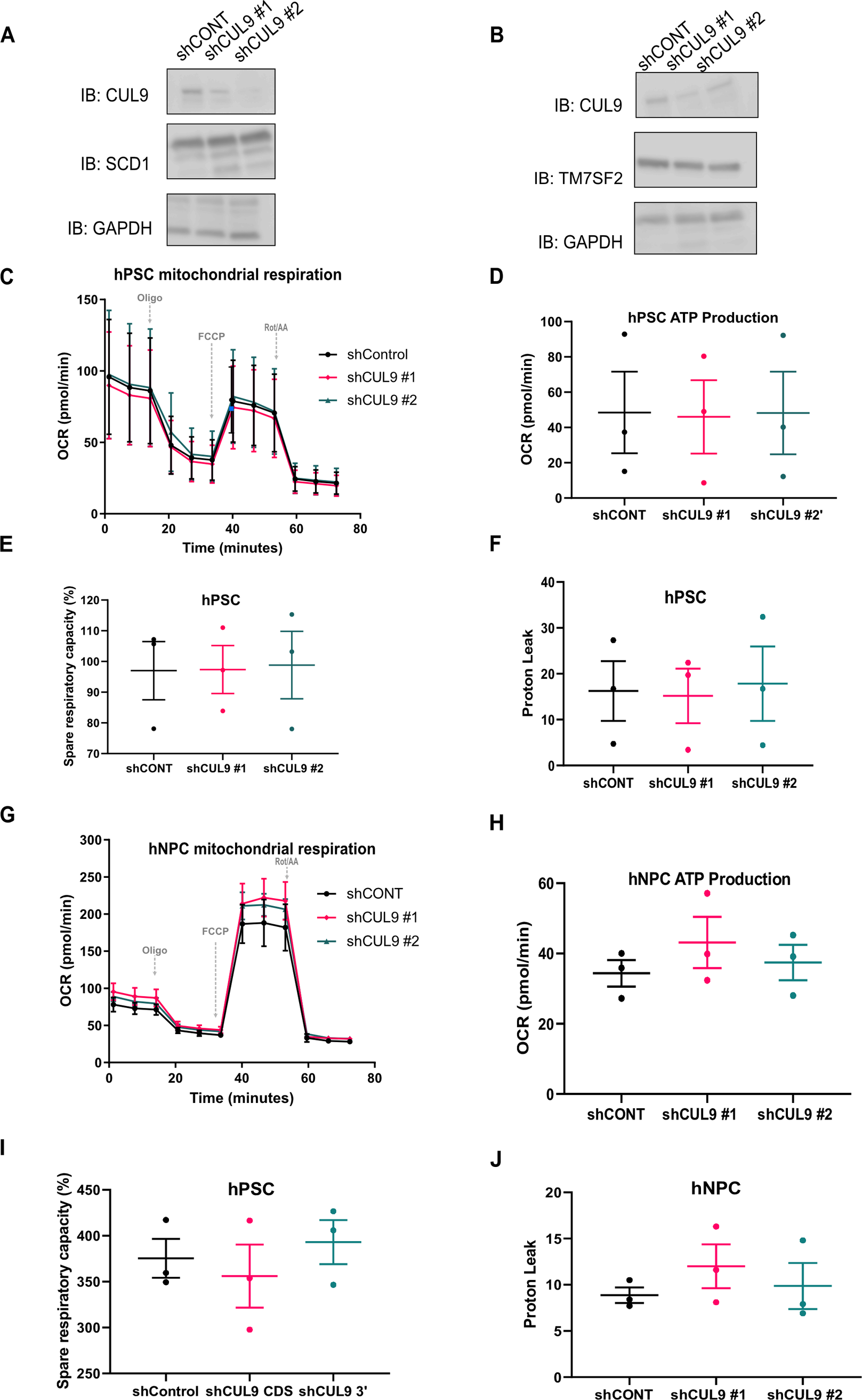

Supplement: S11 Fig — CUL9 KO cells express normal levels of transcription factors CUX1 and SOX3, n = 3, all independent, biological replicates shown in single blot to demonstrate variability between replicates. (TIF) [file pone.0248000.s011.tif]

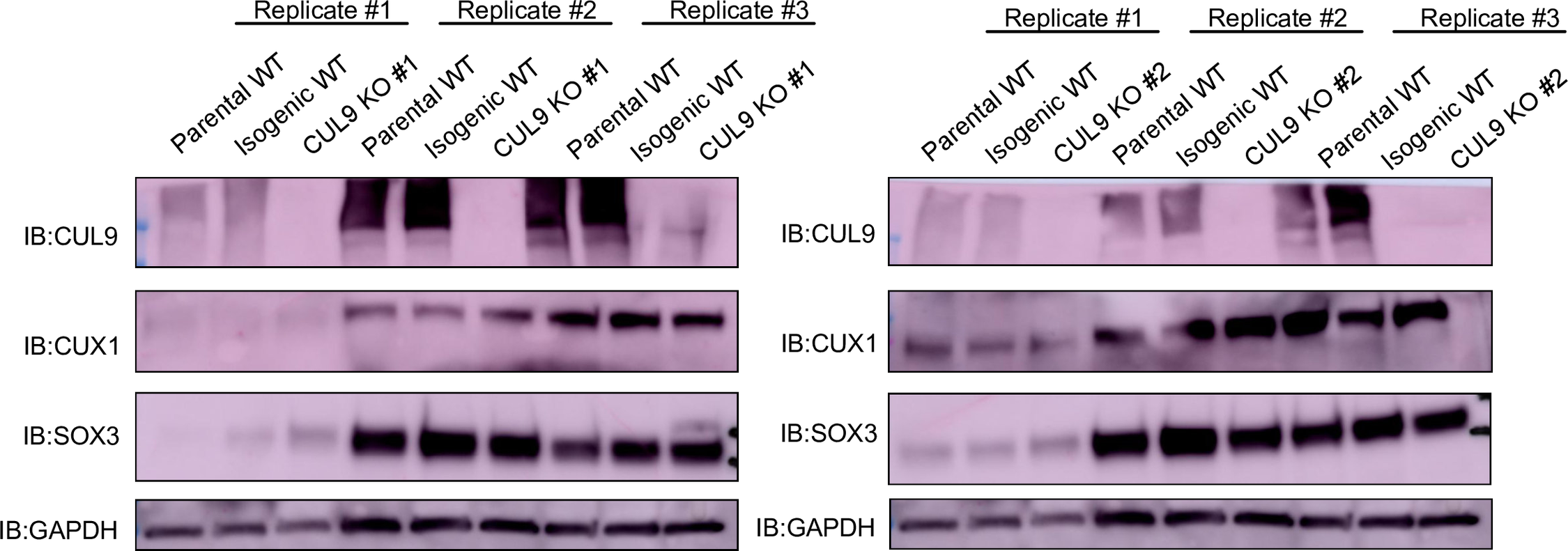

Supplement: S12 Fig — CUL9 KD cells express normal levels of key fatty acid metabolism enzymes SCD1 (A) and DHCR24 (B) n = 3; mean +/- SEM; Analysis done using student’s t-test, α = 0.05. CUL9 KO hPSCs have the same oxygen consumption rate (OCR) (C), ATP production (D), spare respiratory capacity (E), and levels of proton leak (F) as parental and shCONT hPSCs. CUL9 KD hNPCs also display no abnormalities (G-J). n = 3 independent experiments done in triplicate, error bars are +/- SEM. (OCR) was measured using the Seahorse Biosciences Mito Stress Test on an XFe96 analyzer. ATP production was calculated from the corresponding OCR traces in panels A and C for each condition. Spare respiratory capacity is the difference between maximal respiration or basal respiration. Oligomycin inhibits ATP synthase interrupting the electron transport chain ultimately disrupting mitochondrial respiration and ATP production. FCCP is an uncoupler, uncoupling ATP production from the electron transport chain. Rotenone (complex I inhibitor) and antimycin A (complex III inhibitor) completely inhibit mitochondrial respiration, only permitting nonmitochondrial respiration to persist. (TIF) [file pone.0248000.s012.tif]
