# Supplementary material for: A proteomics approach for the identification of cullin-9 (CUL9) related signaling pathways in induced pluripotent stem cell models
Source: PLoS One. 2021 Mar 11;16(3):e0248000. doi: 10.1371/journal.pone.0248000 (PMC7951927; doi:10.1371/journal.pone.0248000)
Supplement: S1 File — (DOCX) [file pone.0248000.s020.docx]

**Supporting information**

**A proteomics approach for the identification of cullin-9 (CUL9) related signaling pathways in induced pluripotent stem cell models**

Natalya A. Ortolano^1^, Alejandra I. Romero-Morales^1^, Megan L. Rasmussen^1^, Caroline Bodnya^1^, Leigh A. Kline^1^, Piyush Joshi^1^, Jon P. Connelly^4,5^, Kristie L. Rose^2,3^, Shondra Pruett-Miller^4,5^ and Vivian Gama^1,6,*^

^1^Vanderbilt University Department of Cell and Developmental Biology, Nashville, 37240, USA ^2^Vanderbilt University Department of Biochemistry, Nashville, 37240, USA,

^3^Vanderbilt MSRC Proteomics Core, Nashville, 37240, USA

^4^St. Jude Children’s Research Hospital, Department of Cell & Molecular Biology, Memphis, 38105, USA.

^5^St. Jude Children’s Research Hospital, Center for Advanced Genome Engineering, Memphis, USA

^6^Vanderbilt Center for Stem Cell Biology, 37240, USA

^7^Vanderbit Brain Institute, 37240, USA

***Corresponding author

E-mail: [vivian.gama@vanderbilt.edu](mailto:vivian.gama@vanderbilt.edu)

# **Supporting methods**

## E8 Media Preparation

Large-batch preparation of basal media E4 consists of 48 L of DMEM/F12 HEPES (Thermo, Cat. 11330057), 3.072 g of L-Ascorbic acid 2-phosphate sesquimagnesium salt hydrate (Sigma, Cat. A8960), 930.86 μL of sodium selenite solution (0.7mg/mL in PBS) (Sigma, Cat. 214485), 26.04 g of sodium bicarbonate (Sigma, Cat S3817), and sodium chloride (Sigma, S7653). Final pH was adjusted to 7.4. E4 is then filtered, and 100 μL of insulin solution (Sigma Cat. I9278), 500 μL of transferrin solution (R&D Systems, Cat. 2914-HT-001G), 50 μg of FGF2 (Peprotech, Cat. 100-18B), and 1 μg of TGFB1 (Peprotech, Cat. 100-21) were added to make E8.

## Neuronal Differentiation

Differentiation of hESCs into neural stem cells was performed using the STEMdiff Neural Induction Medium (Stem Cell Technologies). NPCs were split every seven to nine days and fed daily. Following the third split, cells were maintained in STEMdiff Neural Progenitor Medium (Stem Cell Technologies). This method was employed for cells analyzed in Figure 5C.

Cortical neuron induction of iPSCs was performed following the dual SMAD protocol published by Chambers et al. 2009, except that 0.4 μM LDN193189 (Stemgent Cat. 04-0074) was substituted for Noggin (Chambers et al., 2009; Di Pardo et al., 2017; Neely et al., 2012). Neural induction media consists of 410 mL of Knockout DMEM/F12 (Invitrogen #12660), 75 mL Knockout Serum (Invitrogen #10828), 5 μL of B-mercaptoethanol (Sigma #M3148), and N2 medium. N2 medium consists of DMEM/F12 (Invitrogen #10565-018, + glutamax), 0.775 g D-glucose, and 5mL N2 supplement (Thermo Fisher Scientific #17502048). After 10 days of neural induction, neural differentiation was initiated as reported by Shi et al., 2012. Details and validation of this differentiation method have been previously reported (Di Pardo et al., 2017; Joshi et al., 2019; Neely et al., 2012). Schematic outlining method is in Figure 2A.

## Neural rosette differentiation

Cells were dissociated into single cell suspension and seeded 3.0*10^6^ cells/well of an AggrewellTM 800 (STEM CELL Technologies) in dual SMAD inhibitor media to form neutralized EBs. EBs were incubated at 37°C with 5% CO2 for 48 hours. 50% of total media volume was changed every two days to minimize disruption. On day five, EB diameter was measured using ImageJ, and EBs were harvested according to the manufacturer’s protocol (STEM CELL Technologies) and transferred to a 35mm imaging plate (Cellvis, cat # D35-14-1.5-N) coated with Matrigel (Corning). Media was changed daily through day nine. Cells were fixed with 100% ice-cold methanol (Fisher Scientific, cat # A454-4). Images were acquired using an Andor DU-897 EMCCD camera mounted on a Nikon Spinning Disk microscope equipped with a 0.45 NA 10X and 0.75 NA 20X objectives.

A neural rosette quantification macro was developed using NIS elements to quantify the rosette lumenal area based on ZO-1 staining. Max projections were generated per ROI, followed by advanced denoise of the image. Binary mask intensity thresholding for the ZO-1 was done using control images. Measured data was exported to an Excel file. Data was analyzed in GraphPad Prism using a one-way ANOVA. Outliers were removed prior to ANOVA analysis using ROUT outlier identification method (Q=1%).

## LC-MS/MS analysis

For in-gel digestion, the samples were solubilized in lithium dodecyl sulfate (LDS) sample buffer (Invitrogen, Carlsbad,CA, USA). The samples were loaded onto 4–12% NuPAGE Novex Bis-Tris gel and MOPs running buffer was used for separation (Invitrogen). The samples were allowed to run into the gel for 1.5–2 cm. The gel was fixed and stained with Colloidal Blue Staining kit (Invitrogen) then washed and the stained area was excised and diced to 1 mm cubes. Proteins were reduced with 45 mM DTT for 20 min at 551C, followed by alkylation with 100 mM iodoacetamide for 30 min at room temperature in the dark. The volume of iodoacetamide added was the same as the volume of DTT added. After reduction and alkylation, gel pieces were destained with three consecutive washes with a 50:50 mixture of 50 mM ammonium bicarbonate and ACN for 10 min, dehydrated with 100% ACN for 10 min, and dried in a SpeedVac. The gel pieces were rehydrated in a 50 mL of solution containing 10 ng/mL trypsin (Promega, Madison, WI, USA) in 25 mM ammonium bicarbonate (pH 8.0) for 15 min. 100 mL of 25 mM ammonium bicarbonate buffer was added to each sample and the samples were incubated at 371C for 18 h.

Peptides were extracted using 60% ACN/0.1% TFA twice. The extracted samples were pooled and dried in a SpeedVac and reconstituted in 0.1% formic acid for subsequent analysis.

An analytical column was packed with 20cm of C18 reverse phase material (Jupiter, 3 μm beads, 300Å, Phenomenox) directly into a laser-pulled emitter tip. The peptide solutions were loaded on the capillary reverse phase analytical column (360 μm O.D. x 100 μm I.D.) using a Dionex Ultimate 3000 nanoLC and autosampler. The mobile phase solvents consisted of 0.1% formic acid, 99.9% water (solvent A) and 0.1% formic acid, 99.9% acetonitrile (solvent B). Peptides were gradient eluted at a flow rate of 350 nL/min. The 95 minute gradient consisted of the following: 1-98 min, 2-45% B; 98-105 min, 45-90% B; 105-107 min, 90% B; 107-110 min, 90-2% B; 110-120 min (column equilibration), 2% B. A Q Exactive Plus mass spectrometer (Thermo Scientific), equipped with a nanoelectrospray ionization source, was used to mass analyze the eluting peptides. The instrument method consisted of MS1 using an MS AGC target value of 3e6, followed by up to 15 MS/MS scans of the most abundant ions detected in the preceding MS scan. A maximum MS/MS ion time of 40 ms was used with a MS2 AGC target of 1e5, a 3% underfill ratio and an intensity threshold of 7.5e4. Dynamic exclusion was set to 20s, HCD collision energy was set to 27 and peptide match and isotope exclusion were enabled. For identification of peptides, tandem mass spectra were searched with Sequest (Thermo Fisher Scientific) against a human subset database created from the UniprotKB protein database (www.uniprot.org). Variable modifications of +57.0214 on Cys (carbamidomethylation) and +15.9949 on Met (oxidation) were included for database searching. Search results were assembled using Scaffold 4.3.4. (Proteome Software).

## Quantitative proteomics analysis

Samples were diluted with 100mM TEAB, reduced with 5uL of 200mM TCEP at 55°C for one hour, and available Cys residues were carbamidomethylated with 5μL of 375mM Iodoacetamide for 30 minutes in the dark at room temperature. Protein samples were precipitated with acetone at -20°C. A volume of ice-cold acetone six times the sample volume was added to each sample, and precipitation was carried out overnight. After precipitation, samples were centrifuged at 18,000xg at 4°C, protein pellets were washed with ice-cold acetone, centrifuged, and precipitates were allowed to dry. Proteins were then reconstituted in 100 mM TEAB (pH 8.0) and digested with Promega Gold trypsin overnight at 37°C. Quantitative proteomics analysis was performed using isobaric tags for relative and absolute quantification (iTRAQ). Peptides were labeled with iTRAQ reagents according to the manufacturer’s instructions (SCIEX). After labeling was complete, labeled peptides were combined and fractionation was performed using the Thermo Scientific Pierce High pH Reversed-Phase Peptide Fractionation Kit (Product No. 84868) similar to the manufacturer’s recommended protocol. After loading peptides onto the reverse phase resin, a wash was performed with 5% acetonitrile, 0.1% triethylamine. Elution steps consisted of the following: 7.5%, 10%, 12.5%, 15%, 17.5%, 20%, 22.5%, 25%, 27.55, 30%, 35%, 60%, and 80% acetonitrile with 0.1% triethylamine. Fractions were dried via vacuum centrifugation in SpeedVac concentrator, and peptides were reconstituted in 0.1% formic acid for analysis by LC-coupled tandem mass spectrometry (LC-MS/MS). An analytical column was packed with 35cm of C18 reverse phase material (Jupiter, 3 μm beads, 300Å, Phenomenox) directly into a laser-pulled emitter tip. Peptides were loaded on the capillary reverse phase analytical column (360 μm O.D. x 100 μm I.D.) using a Dionex Ultimate 3000 nanoLC and autosampler. The mobile phase solvents consisted of 0.1% formic acid, 99.9% water (solvent A) and 0.1% formic acid, 99.9% acetonitrile (solvent B). For the first ten fractions, peptides were gradient-eluted at a flow rate of 350 nL/min, using a 180-minute gradient. The gradient consisted of the following: 5-30 %B in 140 min, 30-50 %B in 10 min, 50-70 %B in 5 min, 70 %B in 5 min; 70-5 %B in 4 min, followed by column equilibration for the next sample. A Q Exactive Plus mass spectrometer (Thermo Scientific), equipped with a nanoelectrospray ionization source, was used to mass analyze the eluting peptides. The Q Exactive instrument was operated in data-dependent mode acquiring HCD MS/MS scans (R = 17,500) after each MS1 scan on the 15 most abundant ions using an MS1 ion target of 3 × 106 ions and an MS2 target of 1 × 105 ions. The HCD-normalized collision energy was set to 30, dynamic exclusion was set to 30 s, and peptide match and isotope exclusion were enabled. For the final three fractions, the peptides were combined for LC-MS/MS analysis. This final combined pool of peptides and the 5% acetonitrile wash were analyzed using a similar data acquisition method with a gradient that consisted of 5-50 %B in 105 min, followed by 50-98 %B in 50 min, 98 %B for 5 min, 98-5 %B in 3 min, and column re-equilibration.

Peptide/protein identifications and quantitative analysis were performed using Spectrum Mill (Agilent) as described in Noto et al., 2019. MS/MS spectra were searched against a subset of the UniProt KB protein database containing human proteins. Auto-validation procedures in Spectrum Mill were used to filter the data to <1% false discovery rates at the protein and peptide level. After peptides were identified and proteins were quantified, the results were then filtered to include those proteins for which a minimum of two unique peptides were identified. Log2 protein ratios were fit to a normal distribution using non-linear (least squares) regression. The calculated mean derived from the Gaussian fit was used to normalize individual log2 ratios for each quantified protein. The normalized log2 ratios were then fit to a normal distribution, and the mean and standard deviation values derived from the Gaussian fit of the normalized ratios were used to calculate p values. Subsequently, p values were corrected for multiple comparisons by the Benjamini-Hochberg method (Noto et al., 2019).

## Lentiviral transduction

To generate lentiviral-mediated CUL9 overexpression of iPSC, iPSC colonies at 50-70% confluency were transduced with pLVX-EF1alpha-IRES-mcherry vector (Takara) containing a CUL9-FLAG sequence (1MOI). Cells were fed with mTESR 24 hours later. 48 hours after initial transduction, m-Cherry positive cells were selected for and plated in StemFlex (Thermo) plus CloneR (StemCell Technologies) in 96-well plates using flow activated cell sorting. After 72 hours, cells were fed again with StemFlex plus CloneR. Until colonies were apparent, cells were fed every other day with StemFlex only. Transduction efficiency was validated by Western blot analysis of recovered colonies. Lentivirus was prepared per manufacturer protocol using Lenti-X packaging single shots (VSV-G) (Takara). Lentiviral concentration was determined using Lenti-X GoStix Plus (Takara). Lentiviral particles obtained from Sigma Aldrich (SHCLNV-NM_015089) were used to produce lentiviral stable KD cells. shRNA #1 (TRCN0000004448) targeted the 3’ sequence: 5’-CCGGTCTGTAGTGCTTCCTGTTTGCCTCGAGGCAAACAGGAAGCACTACAGATTTTT-3’ and shRNA #2 (TRCN0000424584) targeted the CDS sequence: 5’- CCGGGATCTCTGTGTCCGTGGAAATCTCGAGATTTCCACGGACACAGAGATCTTTTTTG -3’. Titer provided by Sigma was used to calculate MOI. Cells transduced at 1 MOI.

## CaspaseGlo 3/7 Assay

Cells were plated onto Matrigel (Corning) white round-bottomed 96-well plates at 10,000 cells/well. Triplicate wells were used per condition. After 24 hrs, media was removed and CaspaseGlo 3/7 reagent (Promega #8091) and fresh media was added at a 1:1 ratio. The samples were incubated at room temperature in the dark for one hour. Samples were then analyzed in a Promega GloMax luminometer according to manufacturer instructions.

# **References**

Chambers, S.M., Fasano, C.A., Papapetrou, E.P., Tomishima, M., Sadelain, M., Studer, L., 2009. Highly efficient neural conversion of human ES and iPS cells by dual inhibition of SMAD signaling. Nat. Biotechnol. 27, 275–280. https://doi.org/10.1038/nbt.1529

Di Pardo, A., Amico, E., Basit, A., Armirotti, A., Joshi, P., Neely, M.D., Vuono, R., Castaldo, S., Digilio, A.F., Scalabrì, F., Pepe, G., Elifani, F., Madonna, M., Jeong, S.K., Park, B.-M., D’Esposito, M., Bowman, A.B., Barker, R.A., Maglione, V., 2017. Defective Sphingosine-1-phosphate metabolism is a druggable target in Huntington’s disease. Sci. Rep. 7, 5280. https://doi.org/10.1038/s41598-017-05709-y

Joshi, P., Bodnya, C., Ilieva, I., Neely, M.D., Aschner, M., Bowman, A.B., 2019. Huntington’s disease associated resistance to Mn neurotoxicity is neurodevelopmental stage and neuronal lineage dependent. Neurotoxicology 75, 148–157. https://doi.org/10.1016/j.neuro.2019.09.007

Neely, M.D., Litt, M.J., Tidball, A.M., Li, G.G., Aboud, A.A., Hopkins, C.R., Chamberlin, R., Hong, C.C., Ess, K.C., Bowman, A.B., 2012. DMH1, a Highly Selective Small Molecule BMP Inhibitor Promotes Neurogenesis of hiPSCs: Comparison of PAX6 and SOX1 Expression during Neural Induction. ACS Chem. Neurosci. 3, 482–491. https://doi.org/10.1021/cn300029t

Noto, J.M., Rose, K.L., Hachey, A.J., Delgado, A.G., Romero-Gallo, J., Wroblewski, L.E., Schneider, B.G., Shah, S.C., Cover, T.L., Wilson, K.T., Israel, D.A., Roa, J.C., Schey, K.L., Zavros, Y., Piazuelo, M.B., Peek, R.M., 2019. Carcinogenic Helicobacter pylori Strains Selectively Dysregulate the In Vivo Gastric Proteome, Which May Be Associated with Stomach Cancer Progression. Mol. Cell. Proteomics MCP 18, 352–371. https://doi.org/10.1074/mcp.RA118.001181

Shi, Y., Kirwan, P., Livesey, F.J., 2012. Directed differentiation of human pluripotent stem cells to cerebral cortex neurons and neural networks. Nat. Protoc. 7, 1836–1846. https://doi.org/10.1038/nprot.2012.116
