# Supplementary material for: A proteomics approach for the identification of cullin-9 (CUL9) related signaling pathways in induced pluripotent stem cell models
Source: PLoS One. 2021 Mar 11;16(3):e0248000. doi: 10.1371/journal.pone.0248000 (PMC7951927; doi:10.1371/journal.pone.0248000)

Figure 1C

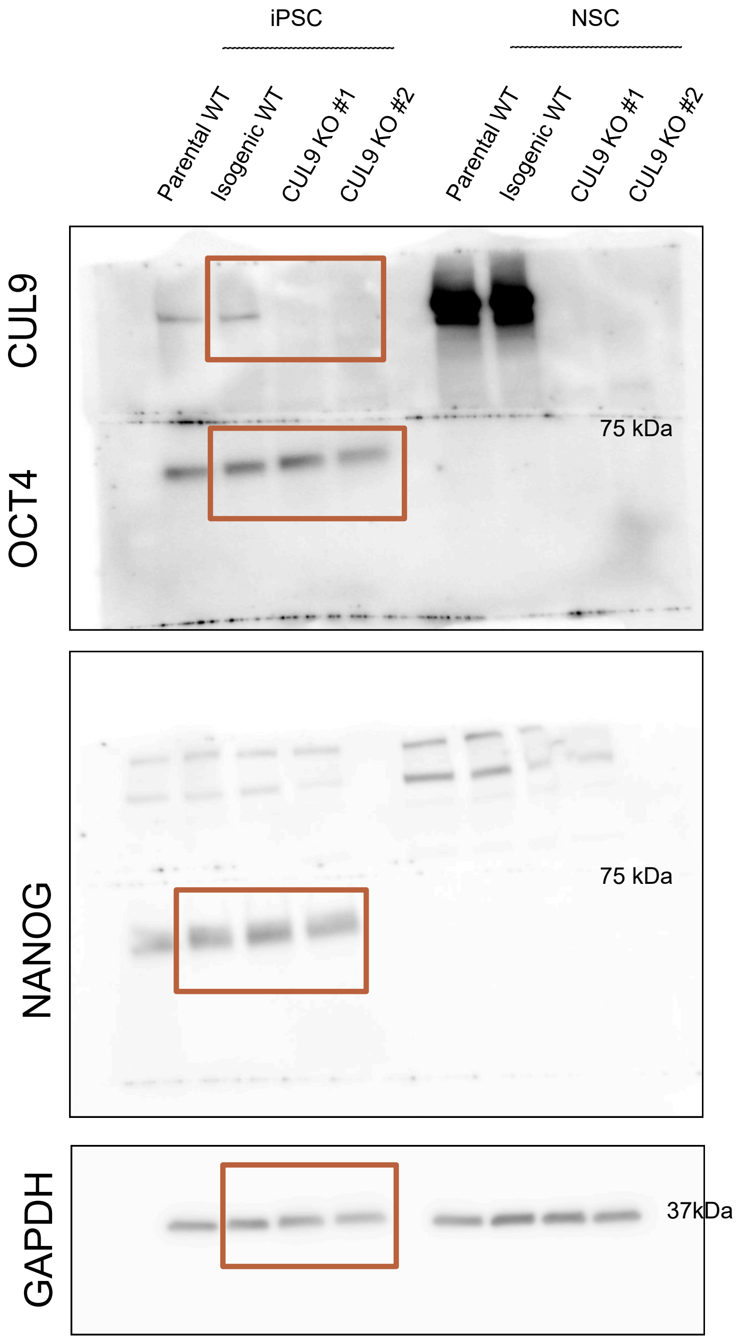

Figure 4B (hPSCs)

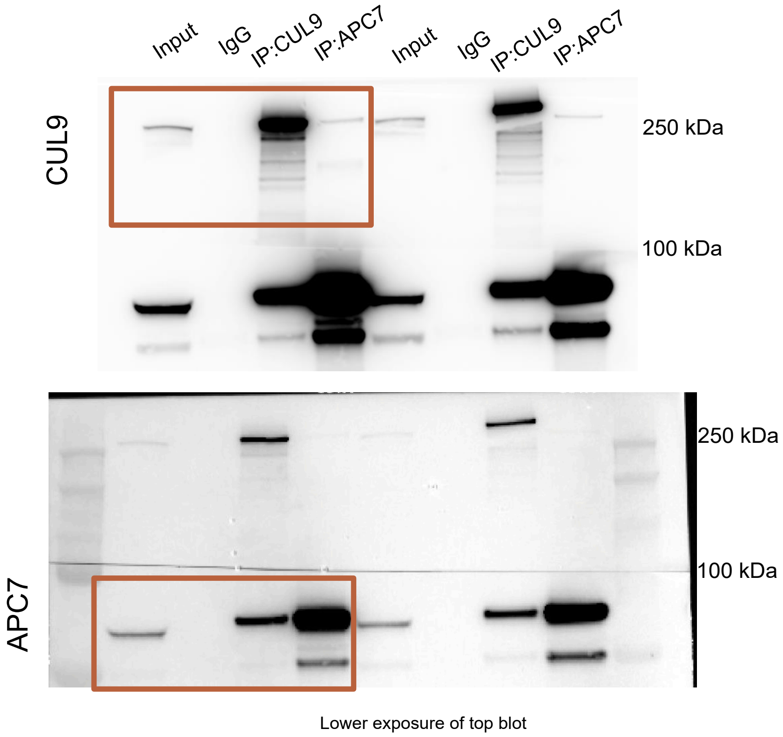

Figure 4B (hNSCs)

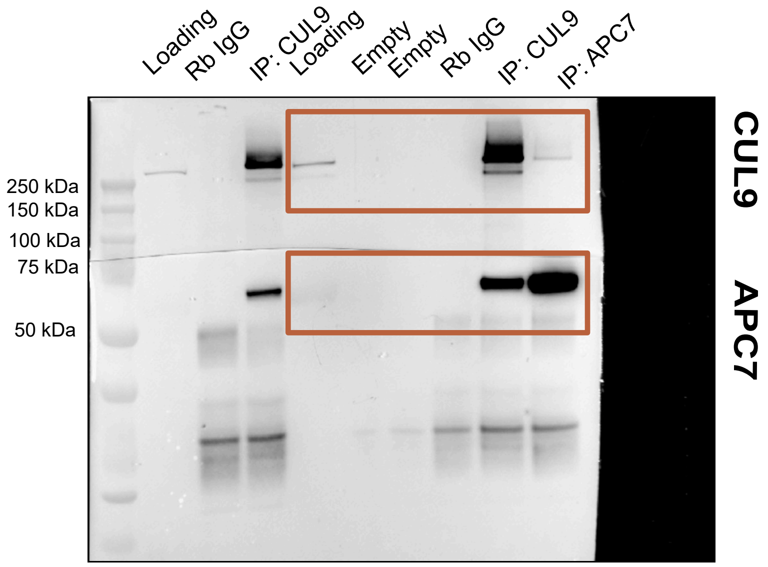

Figure 2B

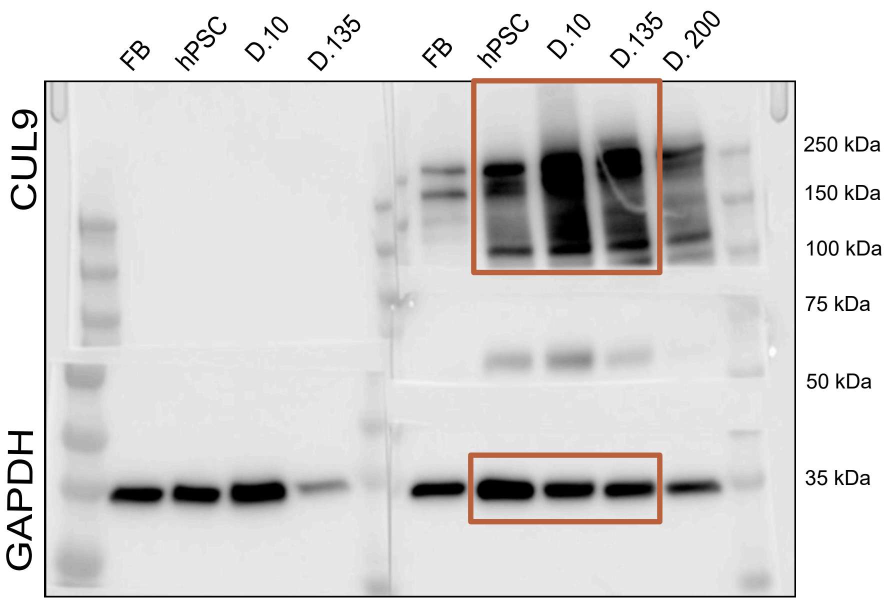

Figure 5A

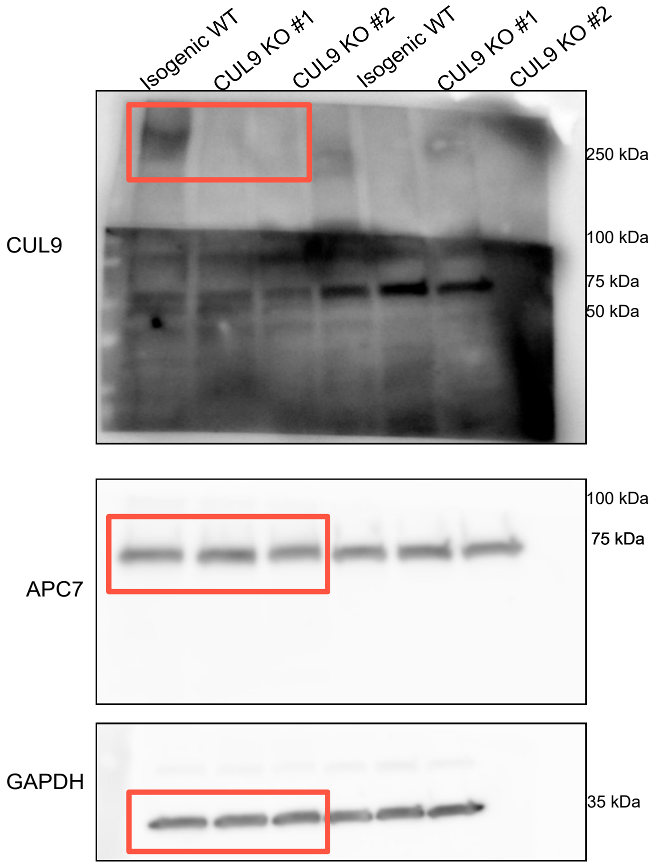

Figure 5B

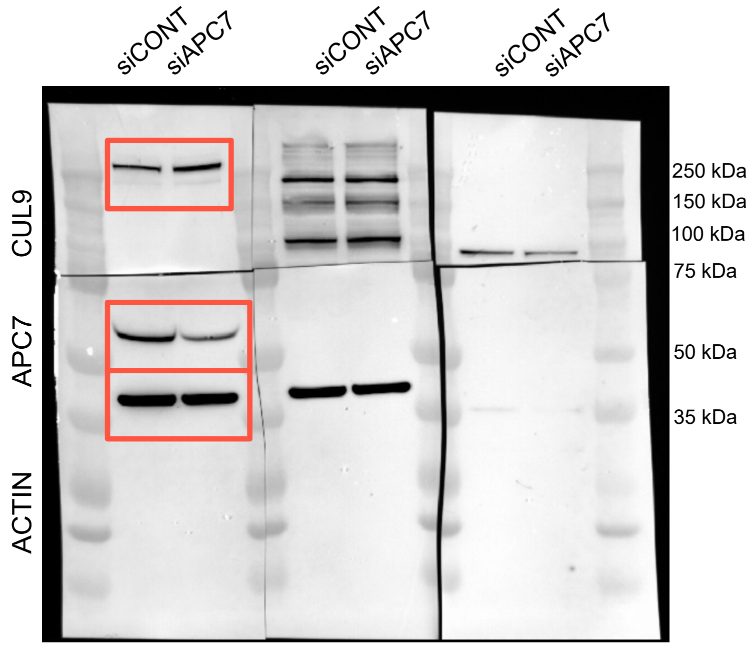

FIgure 5C

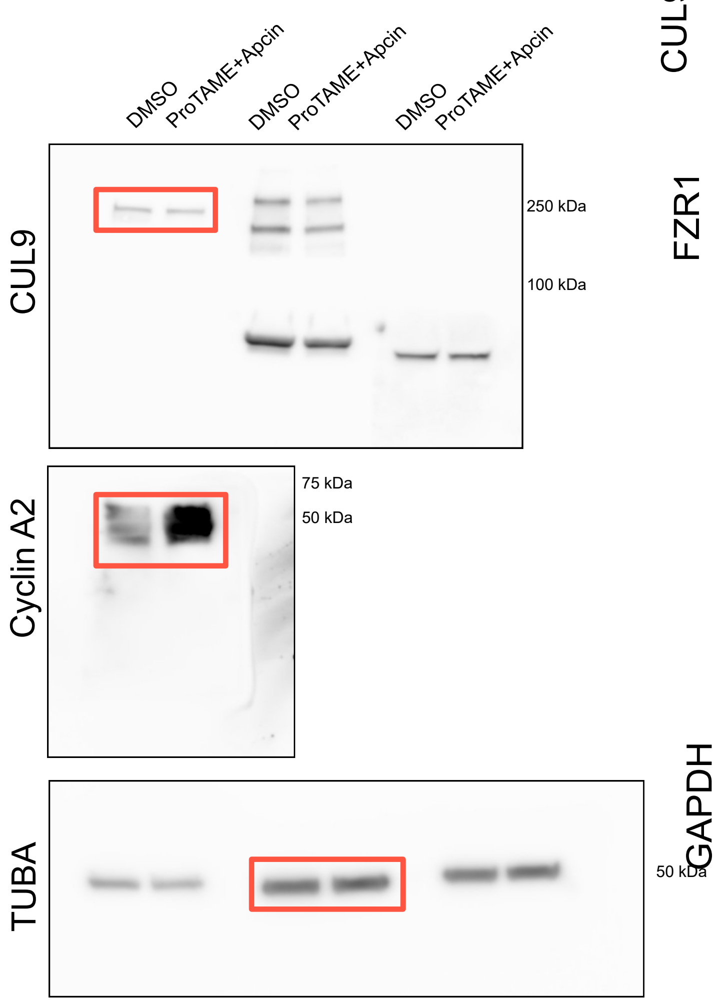

Figure 5D

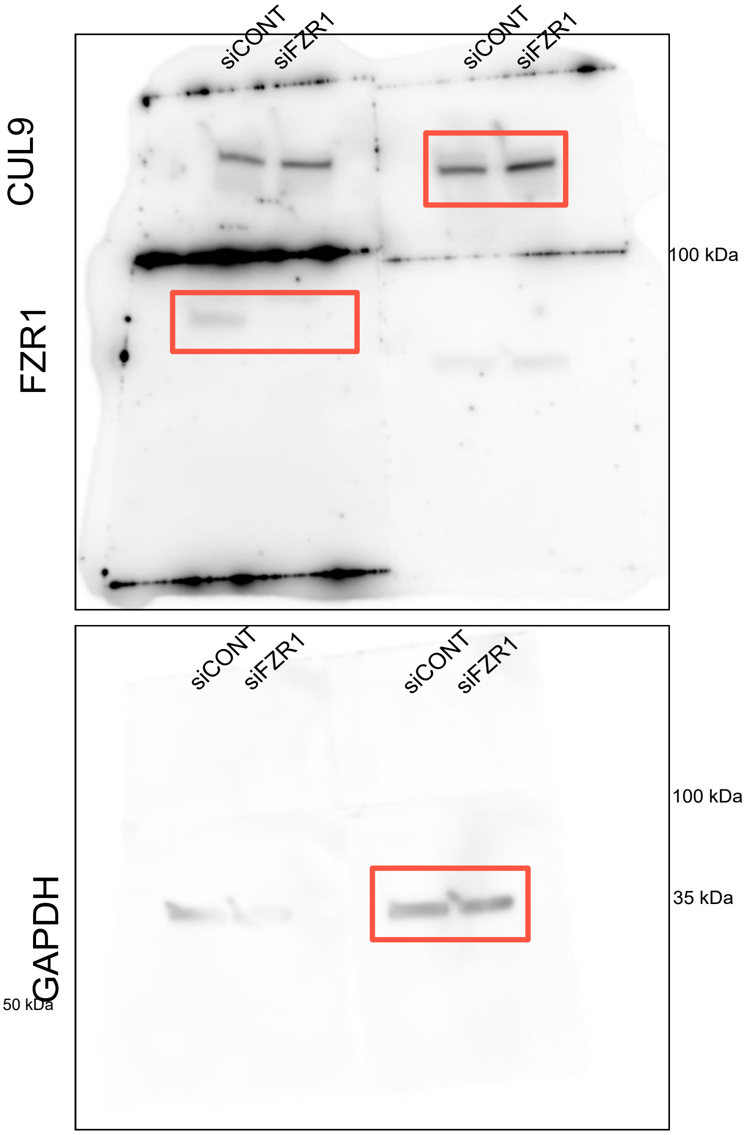

Figure 6B

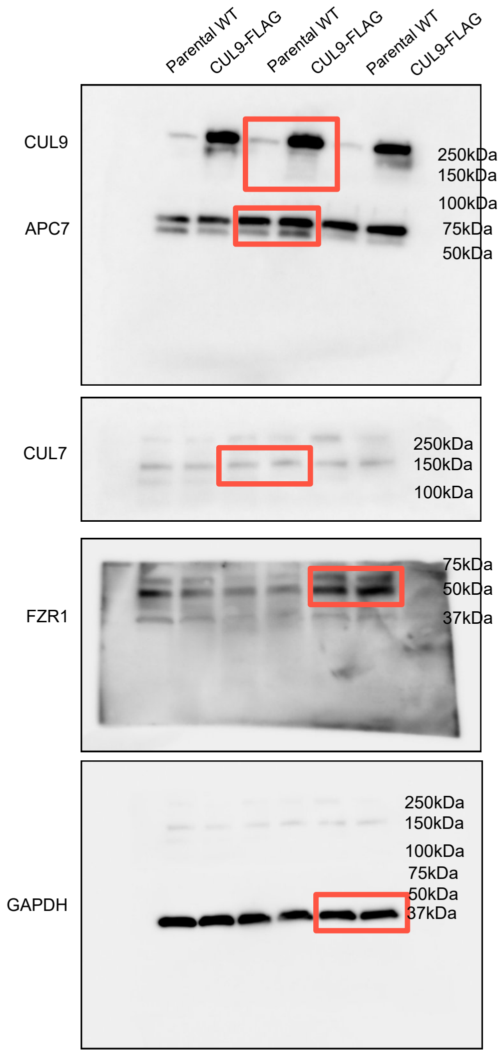

Supp Figure 1C

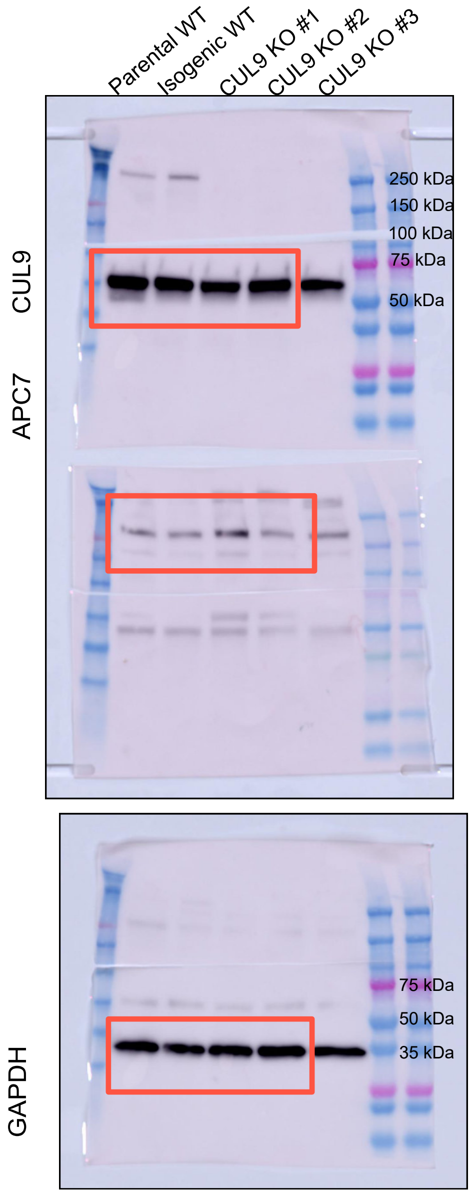

Supp Figure 5A

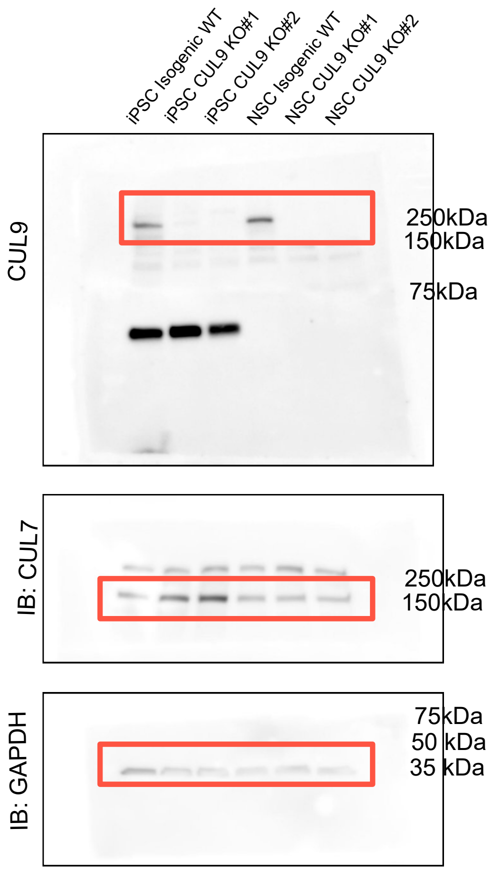

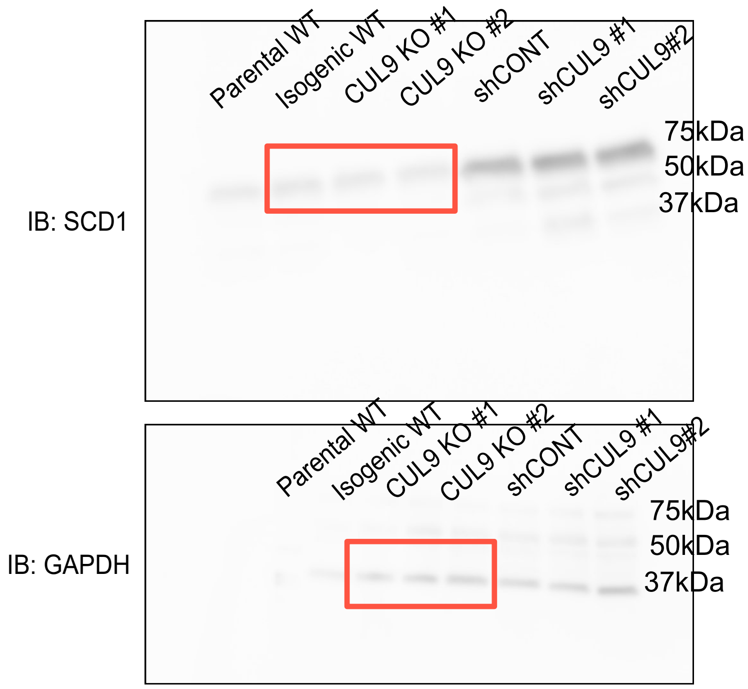

Figure 8 A+B

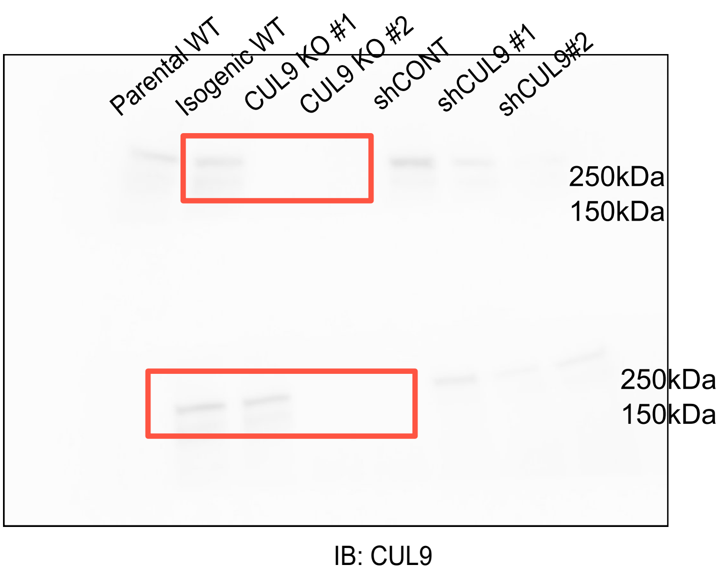

Supp FIgure 5B

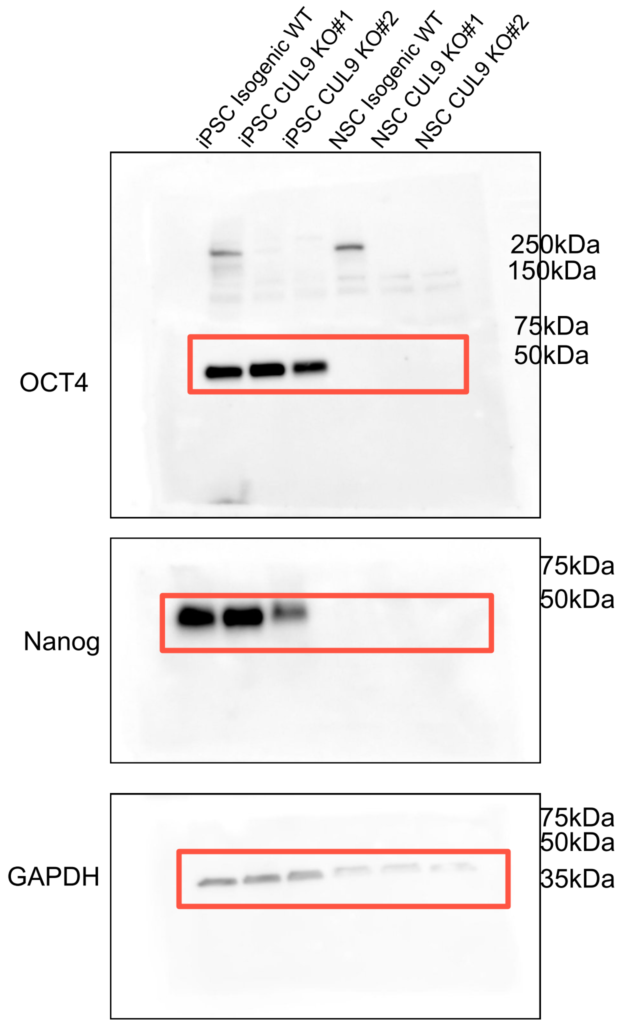

Supp FIgure 5C

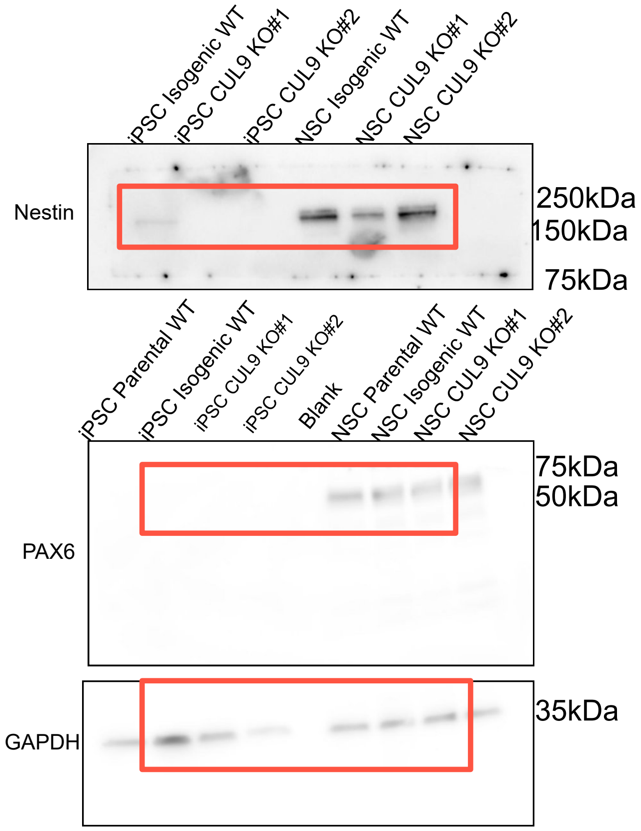

Supp FIgure 6A

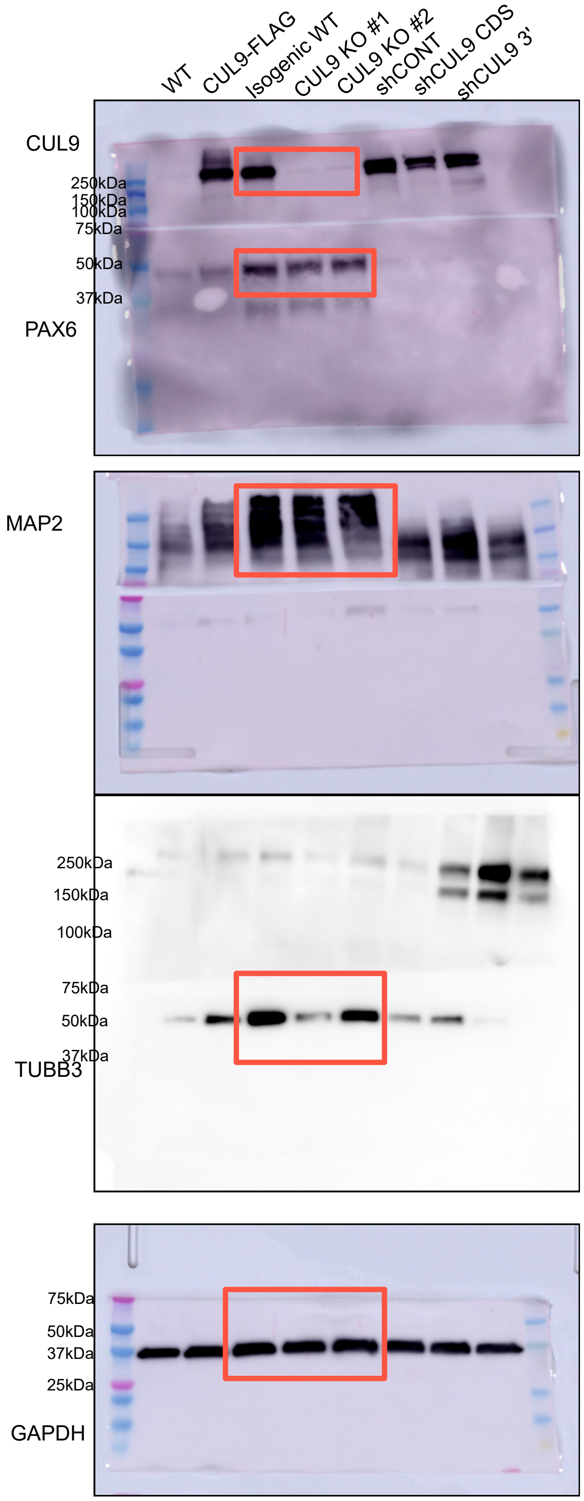

Supp FIgure 7A

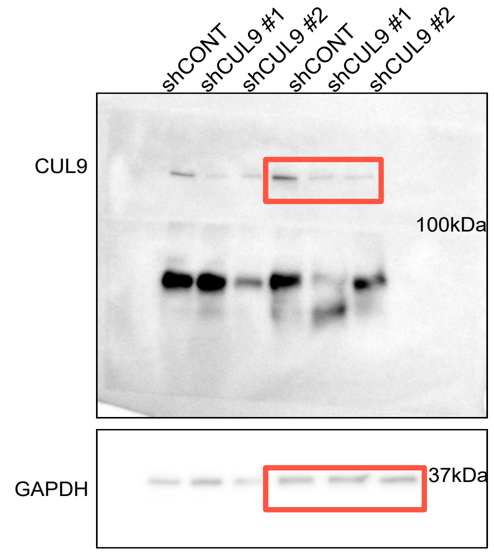

Supp. Figure 10E

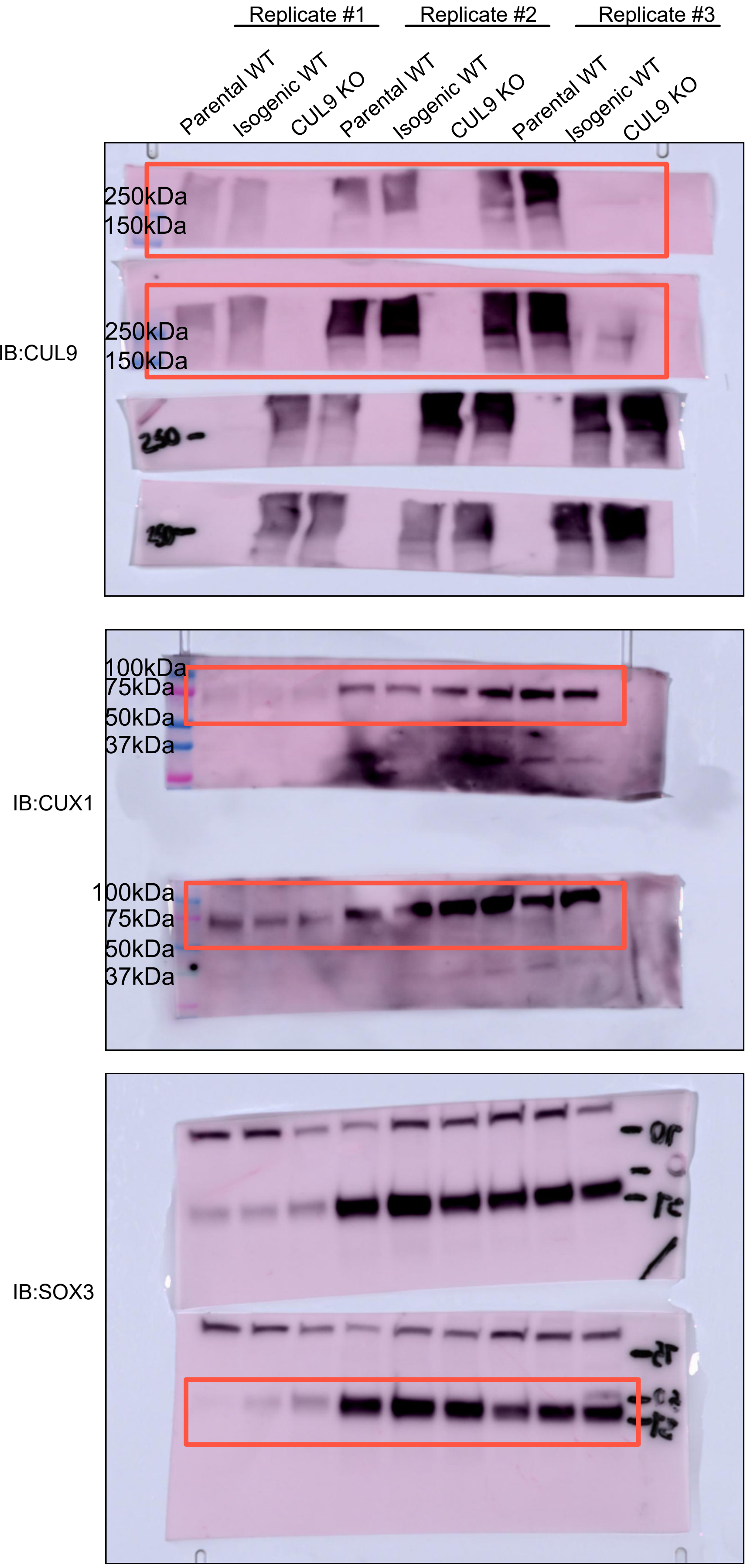

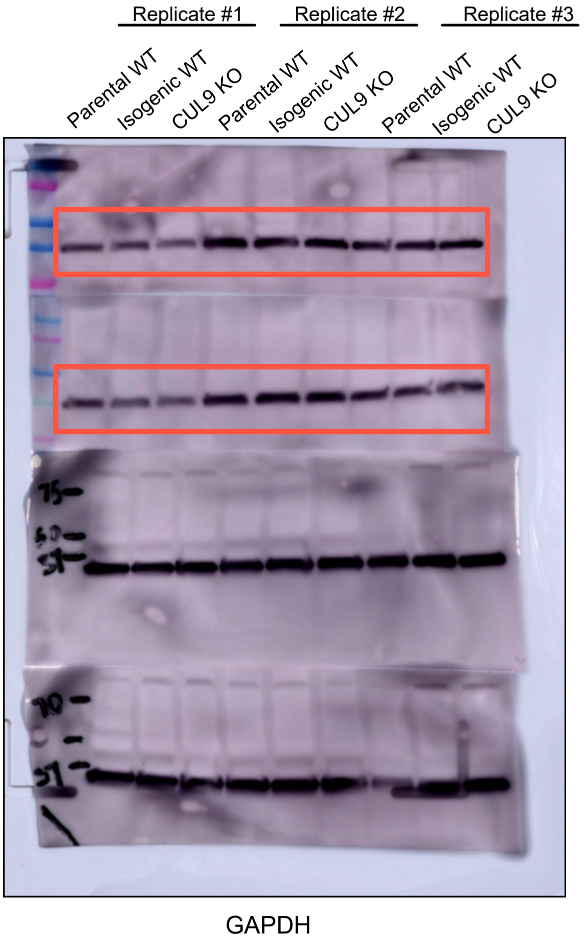

Supp Figure 11A+B

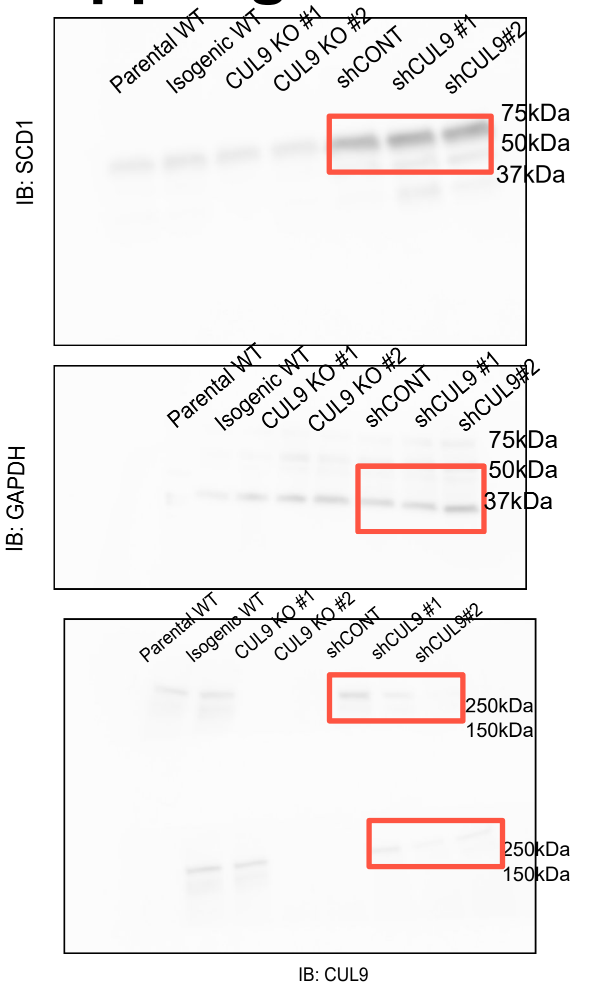

Supplement: S1 Raw images — (PDF) [file pone.0248000.s021.pdf]
